# Population genetic structure and evolutionary genetics of *Anopheles sinensis* based on knockdown resistance (*kdr*) mutations and mtDNA-COII gene in China–Laos, Thailand–Laos, and Cambodia–Laos borders

**DOI:** 10.1186/s13071-022-05366-9

**Published:** 2022-06-26

**Authors:** Yilong Zhang, Canglin Zhang, Linbo Wu, Chunhai Luo, Xiaofang Guo, Rui Yang, Yilong Zhang

**Affiliations:** 1 Department of Tropical Diseases, Faculty of Naval Medicine, Naval Medical University, Shanghai, 200433 China; 2 Yunnan Institute of Parasitic Diseases, Yunnan Provincial Key Laboratory of Vector-Borne Diseases Control and Research, Yunnan Provincial Center of Malaria Research, Yunnan Provincial Collaborative Innovation Center for Public Health and Disease Prevention and Control, Yunnan Institute of Parasitic Diseases Innovative Team of Key Techniques for Vector Borne Disease Control and Prevention (Developing), Pu’er, 665099 China

**Keywords:** Voltage-gated sodium channel gene, Mitochondrial DNA, Evolution, Mutation, Knockdown resistance, *Anopheles sinensis*

## Abstract

**Background:**

Vector control is still a pivotal method for preventing malaria, and its potency is weakened by the increasing resistance of vectors to chemical insecticides. As the most abundant and vital malaria vector in Southeast Asia, the chemical insecticide resistance status in *Anopheles sinensis* remains elusive in Laos, which makes it imperative to evaluate the true nature of chemical insecticide resistance-associated genetic mutations in *An. sinensis* in Laos.

**Methods:**

Adult *An. sinensis* were collected from three border regions in Laos. DNA was extracted from individual mosquitoes. PCR amplification and DNA sequencing of a fragment containing codon 1014 of the voltage-gated sodium channel (*vgsc*) gene were completed to study the *kdr* allele frequency distribution, *kdr* intron polymorphism, population genetic diversity, and the evolutionary status of the *kdr* codon. The mitochondrial cytochrome c oxidase subunit II gene (*COII*) was amplified and sequenced to examine population variations, genetic differentiation, spatial population structure, population expansion, and gene flow patterns.

**Results:**

Nine wild *kdr* haplotypes of the *vgsc* gene were detected in this study, and eight of them, namely 1014L1, 1014L2, 1014L4, 1014L7, 1014L9, 1014L10, 1014L11, and 1014L21, were discovered in the China–Laos border (northern Laos), while 1014L3 was only detected in the Thailand–Laos border (northwestern Laos) and Cambodia–Laos border (southern Laos). The newly identified haplotype, 1014L21, was uniquely distributed in the China–Laos border and was not identified in other countries. Based on sequence analysis of the mitochondrial *COII* genes, significant genetic differentiation and limited gene flow were detected between the China–Laos and Cambodia–Laos *An. sinensis* populations, which suggested that those two regions were genetically isolated. The distinct distribution of the *kdr* haplotype frequencies is probably the result of geographical isolation in mosquito populations.

**Conclusions:**

Lack of *kdr* mutations in the *vgsc* gene was probably due to genetic isolation and the absence of intense selection pressure in the three border regions of Laos. This study reveals that pyrethroid-based chemical insecticides are still appropriate for battling *An. sinensis* in parts of Laos, and routine monitoring of chemical insecticide resistance should be continuously implemented and focused on more restricted areas as part of chemical insecticide resistance management.

**Graphical Abstract:**

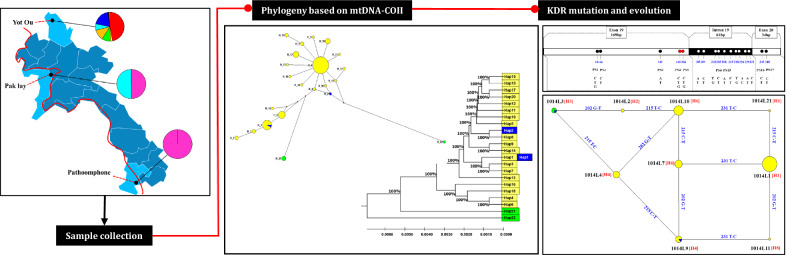

**Supplementary Information:**

The online version contains supplementary material available at 10.1186/s13071-022-05366-9.

## Background

Malaria is a potentially fatal vector-borne disease in tropical and subtropical regions, with 241 million case reports and 627,000 deaths documented worldwide in 2020 [1]. The malaria burden in the Greater Mekong Subregion (GMS) remains a major public health threat affecting the health and lives of a large proportion of people [2]. In the Lao People’s Democratic Republic (Lao PDR), malaria is endemic, whereas the transmission intensity is heterogeneous, with the more intense transmission in remote and forested regions, especially in the southern part of the country [3–5]. Even though Laos has decreased malaria prevalence by 50% since 2000, reoccurrence has been documented since 2011, with over 260,000 patients recorded in 2015 [2]. In recent years, there has been an increase in the number of *Plasmodium vivax* reports in Lao PDR (nearly 50% of *Plasmodium* species from indigenous cases in 2018), although a large proportion of the reported cases of infection have long been related to *Plasmodium falciparum* [4]. Chemical control of vectors has played an essential role in controlling and eliminating malaria [6]. In Laos, as in the majority of GMS nations, malaria vector control depends on the utilization of insecticide-treated materials (i.e., long-lasting insecticide-treated nets [LLIN]) and indoor residual spraying (IRS) [2]. Before using insecticide-treated bed nets (ITNs), residual spraying with DDT (organochlorine family) was employed to prevent malaria. The utilization of DDT was terminated in 1990 (formally abolished in 2010, [7]), and from then on, insecticides based on pyrethroids (e.g., permethrin, deltamethrin, alpha-cypermethrin, and lambda-cyhalothrin) have been used for IRS and/or ITNs [8].

The voltage-gated sodium channel (VGSC) protein is the major target for pyrethroids and DDT [9]. The spectrum of mutations at the *vgsc* gene is remarkably conservative across insect species, revealing convergent evolution [9]. Despite the controversy, many studies have demonstrated that mutations at codon 1014 of VGSC induce resistance to pyrethroids and DDT in substantial arthropod species [9–11]. The main target-site resistance mechanism in *Anopheles* mosquitoes involves three non-synonymous variants (L1014F, L1014C, and L1014S) at the *kdr* codon L1014 of the *vgsc* gene, causing resistance to pyrethroids [10]. In Asia, those variants have been recorded in certain *Anopheles* populations from India [12, 13], Sri Lanka [14], Indonesia [15], China [16, 17], Vietnam, and Cambodia [18]. In *Anopheles sinensis*, the most abundant and important malaria vector in Southeast Asia [19–23], a remarkable positive association between *kdr* allele frequency and bioassay-based resistance phenotype has been recorded [17, 24–26]. Therefore, *kdr* mutation has been utilized as a molecular marker for monitoring the resistance of pyrethroids in *An. sinensis* [17]. In *An. sinensis*, four non-synonymous variants at codon L1014 of the *vgsc* gene are identified, namely the L1014F [17, 24, 25, 27–29], L1014S [18, 25], L1014C [17, 24, 27–29], and L1014W [25] mutations.

Due to the use of various insecticides in vector control and continuous use in agricultural activities, the geographical distribution and density of malaria vectors may change, and insecticide resistance is also expected [30]. Various factors could influence the distribution of *kdr* allele variants in *An. sinensis* throughout Southeast Asia. The intricacy of landscapes can impede genetic flow between mosquito populations. Selection based upon the intensity and duration of pyrethroid use can determine the allele distribution pattern of *kdr* as well [31]. Additionally, the genetic variation based on neutral biomarkers can reflect the population structure resulting from demographic factors. In this case, the cytochrome *c *oxidase genes of the mitochondria genome are utilized as the neutral reference [31]. Based on its unique features of maternal inheritance, no recombination, high variability compared with nuclear DNA, and low population size required, mitochondrial DNA (mtDNA) has become a popular biomarker for studies on genetic diversity and population structure [32].

The status of chemical insecticide resistance in *An. sinensis* remains elusive in the Lao PDR. Given that vector control is still a pivotal method in preventing malaria and that its potency is attenuated by the increasing resistance of vectors to insecticides, it is imperative to evaluate the actual occurrence of chemical insecticide resistance-related genetic variants in *An. sinensis* in Laos. In this study, the *kdr* allele distribution was investigated in *An. sinensis* adult samples collected from multiple sites, including the China–Laos border, Cambodia–Laos border, and Thailand–Laos border. Additionally, the possible evolutionary origin of *kdr* haplotypes was studied, and the role of the demographic history of *An. sinensis* on the evolutionary process of *kdr* variants was examined using mtDNA sequencing information.

## Methods

### Mosquito collection and identification

Using overnight trapping with battery-operated Centers for Disease Control and Prevention (CDC) light traps (model 1012, John W. Hock Inc., USA) in cattle/pig pens or human rooms from 8:00 pm to 8:00 am, adult mosquitoes were collected in Pathoomphone County (Champasak Province) in 2017, as well as in Pak lay County (Xayabuli Province) and Yot Ou County (Phongsaly Province) in 2019 in accordance with our previous study [33]. The live adult mosquitoes were killed by freezing in a refrigerator. The subsequent morphological identification was carried out by sex, species, and subgroup, using a dissecting microscope with the keys of Das et al. [34]. Each morphologically identified specimen was kept individually in a 1.5-ml microcentrifuge tube with 75% ethanol and stored at 4 °C for molecular species confirmation and further processing.

After that, the molecular identification of *An. sinensis* based on cytochrome c oxidase subunit II (*COII*) was carried out to avoid any variations in further analysis. Ninety-eight percent sequence identity with the voucher specimens/sequences in the NCBI Nucleotide database is required for final species confirmation. To avoid the issue of inadequate results for the voucher sequence produced by *COII* alone, we performed amplification and sequencing on ITS2. Hence, ITS2 and *COII* database comparisons of each morphologically identified *An. sinensis* sample were paired to determine the species of *An. sinensis* in accordance with previous studies [35, 36] (Additional file 1: Table S1).

### DNA extraction, *COII*/*kdr* amplification, and sequencing

A total of 134 morphologically identified *An. sinensis* specimens were further screened for DNA extraction, *COII*/*kdr* amplification, and sequencing (Additional file 1: Table S1), and 89 of them were eventually molecularly identified as *An. sinensis* based on both ITS2 and *COII* (Additional file 1: Table S1). The extraction of genomic DNA in individual mosquitoes was carried out following the manufacturer’s instructions (QIAamp^®^ DNA Mini Kit, Germany). The amplification for approximately 650 bp of the *COII* gene was carried out using primers LEU-F (5′-TCTAATATGGCAGATTAGTGCA-3′) and LYS-R (5′-ACTTGCTTTCAGTCATCTAATG-3′) [33]; a fragment containing codon 1014 of the *An. sinensis* *vgsc* gene (325-bp polymerase chain reaction [PCR] product of the *kdr* gene) was amplified using primers KDR-F (5′-TGCCACTCCGTGTGTTTAGA-3′) and KDR-R (5′-GAGCGATGATGATCCGAAAT-3′) [24]. For amplification of *COII*, the cycling parameter included 95 °C, 5 min; 95 °C/1 min, 51 °C/1 min, 72 °C/2 min for 35 cycles; with a final extension of 72 °C for 10 min. For amplification of KDR, the cycling parameter included 95 °C, 5 min; 94 °C/30 s, 55 °C/30 s, 72 °C/30 s for 35 cycles; with a final extension of 72 °C for 10 min. The PCR mixture (25 μl) consisted of 12.5 μl 2 × Taq PCR Mix (Tiangen Biotech, Beijing, China), 5 μl of template DNA (< 1 μg), 1 μl of 10 μM each primer, and 5.5 μl water. The PCR products were analyzed by 1.5% agarose gel electrophoresis stained with GoldView (Solarbio, China) under ultraviolet (UV) transillumination. PCR products were purified by an agarose gel DNA recovery kit (DP219, Tiangen Biotech, Beijing, China) before sequencing. The sequencing reaction was performed in both directions using an ABI Big Dye Terminator Kit v.3.1 (Applied Biosystems, Warrington, UK) and was analyzed using the ABI Prism 3500xL Genetic Analysis Tool (Applied Biosystems, CA, USA) in Shanghai (Sangon Biotech).

### Sequence alignment and phylogenetic analysis based on *COII*/*kdr* sequences

The researchers used the keywords “(species name) & *COII*/*kdr*” for searching *COII* or *kdr* sequences of *An. sinensis* deposited in GenBank. Additional file 2: Table S2 shows the mentioned sequences of *COII*, while *kdr* sequences are listed in Table 1. The *COII* and *kdr* sequence data set was combined with our original data and records retrieved from GenBank. A multiple sequence alignment was conducted in MEGA-X [37], and the manual adjustment was conducted using BioEdit V7.0.9 if required [38]. Gaps were excluded from the analysis, and characters were unweighted. A phylogenetic tree was generated using a neighbor-joining algorithm bootstrapped with 1000 replicates [39] based on MEGA-X [37]. The visualization of this phylogram was performed using FigTree v1.4.2 [40].

**Table 1:** *kdr* haplotypes identified or/and used in this study

Known distribution in Laos and China^a^	Total	Haplotype code	Polymorphic sites	*n*	Intron types	GenBank ID
LA-LPY	83	1014L1 1014L2 1014L4 1014L7 1014L9 1014L10 1014L11 1014L21^b^	CTT**TG***ACGCATGCTT*CC CTT**TG***ACGCACGCTC*TC CTT**TG***ACTCATGCTC*TC CTT**TG***ACGCATGCTC*CC CTT**TG***ACTCATGCTC*CC CTT**TG***ACGCATGCTC*TC CTT**TG***ACTCATGCTT*CC CTT**TG***ACGCATGCTT*TC	39 2 8 10 6 16 1 1	H1 H2 H4 H6 H4 H6 H8 H1	This study
LA-LXP	2	1014L3 1014L9	CTT**TG***ACTCACGCTC*TC CTT**TG***ACTCATGCTC*CC	1 1	H3 H4	This study
LA-LCP	4	1014L3	CTT**TG***ACTCACGCTC*TC	4	H3	This study
CN-GX	22	1014L1 1014L2 1014L3 1014L4 1014L5 1014L6 1014L7 1014L8 1014L9 1014L15 1014L16 1014L17 1014L18 1014F1 1014F2 1014S1 1014S2 1014S3 1014S4 1014S5 1014S6 1014C1	CTT**TG***ACGCATGCTT*CC CTT**TG***ACGCACGCTC*TC CTT**TG***ACTCACGCTC*TC CTT**TG***ACTCATGCTC*TC GTT**TG***ACGCACGCTC*TC CTT**TG***TCGCACGCTC*TC CTT**TG***ACGCATGCTC*CC CTT**TG***ATGCACGCTC*TC CTT**TG***ACTCATGCTC*CC CTT**TG***ACGCATGATT*CC CTT**TG***ACTTACGCTC*TC TTT**TG***ACGCATGCTC*CT CTT**TG***ACGCACTCTC*TC CTT**TT***ACGCATGCTT*CC CTT**TT***ACTCACGCTC*TC GTT**CG***ACGCACGCTC*TC CTT**CG***ACGCACGCTC*TC CTT**CG***ACTCACGCTC*TC CTT**CG***ACTCATGCTC*TC CTT**CG***ACGCATGCTT*CC CTT**CG***TCGCACGCTC*TC CTT**GT***ACGCATGCTT*CC	1 1 1 1 1 1 1 1 1 1 1 1 1 1 1 1 1 1 1 1 1 1	H1 H2 H3 H4 H2 H5 H6 H7 H4 H10 H11 H6 H12 H1 H3 H2 H2 H3 H4 H1 H5 H1	KY014584.1 KY014585.1 KY014586.1 KY014587.1 KY014588.1 KY014589.1 KY014590.1 KY014591.1 KY014592.1 MH384264.1 MH384265.1 MH384266.1 MH384267.1 KY014598.1 KY014599.1 KY014593.1 KY014594.1 KY014595.1 KY014596.1 MH384262.1 MH384263.1 KY014597.1
CN-AH	10	1014L2 1014L14 1014F1 1014F2 1014F4 1014F6 1014F9^b^ 1014C1 1014C2	CTT**TG***ACGCACGCTC*TC CTT**TG***ACGCACGCTT*TC CTT**TT***ACGCATGCTT*CC CTT**TT***ACTCACGCTC*TC CTA**TT***ACTCATGCTT*CC CTA**TT***ACGCATGCTT*CC CTT**TT***ACTCATGCTT*CC CTT**GT***ACGCATGCTT*CC CTT**GT***ACTCATGCTT*CC	1 1 2 1 1 1 1 1 1	H2 H9 H1 H3 H8 H1 H5 H1 H8	KF697678.1 KF697679.1 KF697674.1/KF697681.1 KF697677.1 KF697680.1 KF697676.1 KF697682.1 KF697675.1 KF697683.1
CN-ZJ	10	1014L1 1014L2 1014L3 1014L4 1014L5 1014F1 1014F6 1014F7^b^ 1014F8^b^ 1014C1	CTT**TG***ACGCATGCTT*CC CTT**TG***ACGCACGCTC*TC CTT**TG***ACTCACGCTC*TC CTT**TG***ACTCATGCTC*TC GTT**TG***ACGCACGCTC*TC CTT**TT***ACGCATGCTT*CC CTA**TT***ACGCATGCTT*CC CTT**TT***ACGCACGCTC*TC CTT**TT***ACGCATGCAT*CC CTT**GT***ACGCATGCTT*CC	1 1 1 1 1 1 1 1 1 1	H1 H2 H3 H4 H2 H1 H1 H2 H14 H1	MG953793.1 MG953794.1 MG953792.1 MG953790.1 MG953799.1 MG953791.1 MG953798.1 MG953797.1 MG953796.1 MG953795.1
CN-YN	5	1014L1 1014L2 1014L3 1014L6 1014L13	CTT**TG***ACGCATGCTT*CC CTT**TG***ACGCACGCTC*TC CTT**TG***ACTCACGCTC*TC CTT**TG***TCGCACGCTC*TC CTT**TG***TCGCACGCTC*CC	1 1 1 1 1	H1 H2 H3 H5 H5	KF697669.1 KF697670.1 KF697672.1 KF697671.1 KF697673.1
CN-HaN	11	1014L1 1014L2 1014L3 1014L4 1014L5 1014L6 1014L8 1014L11 1014L19^b^ 1014L20^b^ 1014F7^b^	CTT**TG***ACGCATGCTT*CC CTT**TG***ACGCACGCTC*TC CTT**TG***ACTCACGCTC*TC CTT**TG***ACTCATGCTC*TC GTT**TG***ACGCACGCTC*TC CTT**TG***TCGCACGCTC*TC CTT**TG***ATGCACGCTC*TC CTT**TG***ACTCATGCTT*CC CTT**TG***ACGCTTGCTT*CC CCT**TG***ACGCACGCTC*TC CTT**TT***ACGCACGCTC*TC	1 1 1 1 1 1 1 1 1 1 1	H1 H2 H3 H4 H2 H5 H7 H8 H13 H2 H2	KF718271.1 KF718272.1 KF718269.1 KF718270.1 KF718274.1 KF718275.1 KF718273.1 KP763787.1 KF718277.1 KF718276.1 KF718278.1
CN-HeN	9	1014L1 1014L2 1014L3 1014L6 1014L10 1014F1 1014F5 1014C1 1014W1^b^	CTT**TG***ACGCATGCTT*CC CTT**TG***ACGCACGCTC*TC CTT**TG***ACTCACGCTC*TC CTT**TG***TCGCACGCTC*TC CTT**TG***ACGCATGCTC*TC CTT**TT***ACGCATGCTT*CC CTT**TC***ACGCATGCTC*CC CTT**GT***ACGCATGCTT*CC CTT**GG***ACGCATGCTT*CC	1 1 1 1 1 1 1 1 1	H1 H2 H3 H5 H6 H1 H6 H1 H1	KF927164.1 KF927163.1 KF927160.1 KP763803.1 KF927162.1 KF927157.1 KF927156.1 KF927155.1 KF927159.1
CN-GZ	1	1014L10	CTT**TG***ACGCATGCTC*TC	1	H6	KP763768.1
CN-SC	1	1014L12	CTT**TG***ACTCACGCTC*CC	1	H3	KP763792.1
CN-HuB	1	1014F3	CTT**TC***ACTCACGCTC*TC	1	H3	KP763782.1

^a^Distribution information is adopted from GenBank and this study

^b^The seven haplotypes which were newly identified in other research and the present study. The polymorphic sites within intron 19 are in italics. The bold and underlined letters denote the nonsynonymous mutations on sites 165 and 166. LA-LPY, Yot Ou County (Phongsaly Province); LA-LCP, Pathoomphone County (Champasak Province); LA-LXP, Pak lay County (Xayabuli Province); CN-GX, Guangxi Province (China); CN-GZ, Guizhou Province (China); CN-HaN, Hainan Province (China); CN-HeN, Henan Province (China); CN-SC, Sichuan Province (China); CN-YN, Yunnan Province (China); CN-ZJ, Zhejiang Province (China); CN-AH, Anhui Province (China); CN-HuB, Hubei Province (China)

### Genetic diversity analysis and neutrality test based on *COII*/*kdr* sequences

DnaSP v.5.0 [41] was used to calculate the average of nucleotide differences per site (K), nucleotide diversity (π), haplotype diversity (Hd), and haplotypes (H) based on the *COII* and *kdr* sequences. To compare the *COII* and/or *kdr* haplotypes between Laos and other geographical regions, existing data in GenBank from other countries were also analyzed. The parsimony framework was applied using Network 4.0 [42] on both genes, and Tajima’s *D *[43] and Fu’s *Fs* [44] were calculated for haplotype data using DnaSP v.5.0 [41] to test the hypothesis of strict neutrality in the *An. sinensis* population.

### Statistical analysis based on *COII* sequences

Based on *COII* sequences, Arlequin v.3.5 [45] was used to calculate pairwise *F*_ST_ for estimating population differentiation by complying with a difference in haplotype frequency, Nei’s *Nm* estimated gene flow conformed to GST [46], and analysis of molecular variance (AMOVA) for determining the distribution of genetic variation in the population using 1000 permutations. Isolation by distance (IBD) was examined using a nonparametric Mantel test with the web-based computer program IBDWS v.3.16 [47]. For distinguishing smooth unimodal distribution from multimodal or ragged distribution, the mismatch distribution (simulated in Arlequin v.3.5) was used [48–50].

## Results

### Sequence polymorphisms of the *An. sinensis vgsc* gene

**Fig. 1:**
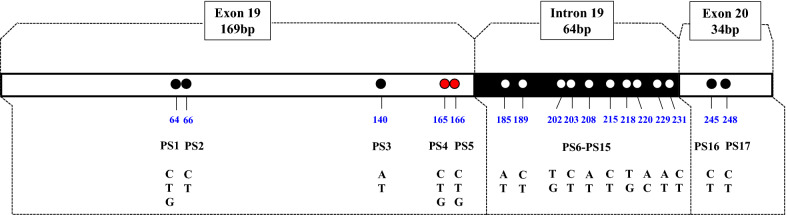
The nucleotide region of the *An. sinensis* *vgsc* gene addressed in this study. Dots indicate the polymorphic sites (PSs) in the obtained sequences. The red dots represent sites leading to nonsynonymous mutations. The positions of PSs in the 267-bp sequence are numbered below the dots. The nucleotides for each PS are given

The 267-bp DNA fragments individually amplified from a total of 89 *An. sinensis* mosquitoes were used for sequence polymorphism analysis. This sequence covered partial exon 19 (contains the codon 1014), intron 19, and partial exon 20 of the *An. sinensis vgsc* gene (KE525266.1). In general, 17 nucleotide polymorphic sites (PSs) were identified. The first to fifth PSs were located on exon 19, the sixth to 15th PSs on intron 19, and the 16th and 17th PSs on exon 20. The polymorphisms in the fourth and fifth PSs resulted in amino acid substitutions (L/F/S) at codon 1014, and the nucleotide variations in the first, second, third, 16th, and 17th PSs represented synonymous mutations (Fig. 1).

### Diversity and frequency of *kdr* haplotypes

**Fig. 2:**
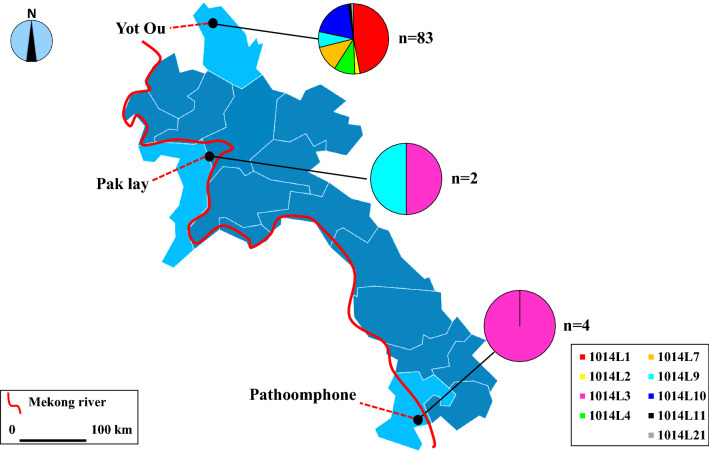
Distribution and frequency of *kdr* alleles in *An. sinensis* populations along the China–Laos border, Thailand–Laos border, and Cambodia–Laos border. Pak lay County (Xayabuli Province); Yot Ou County (Phongsaly Province); Pathoomphone County (Champasak Province). Red line = Mekong River. The shapefile map of Laos was downloaded and prepared by using Pixel Map Generator-Beta online (amCharts, Vilnius, Lithuania) (https://pixelmap.amcharts.com/), which is copyright-free

Point mutations at position 1014 of the domain II of the VGSC protein have been documented to confer *kdr* [9, 10]. To better understand the diversity and frequency of *kdr* haplotypes in this area, we downloaded 70 *kdr* sequences of *An. sinensis* from NCBI, combined with our original data from 89 samples collected in Laos. Thirty-nine haplotypes were identified from 159 *An. sinensis* individuals (Table 1). Seven haplotypes (i.e., 1014L19, 1014L20, 1014L21, 1014F7, 1014F8, 1014F9, 1014W1) were newly identified in other research except for one haplotype, 1014L21, which was newly identified in this study. In the present study, only a wild haplotype of *kdr* was detected, TTG (1014L); other mutations such as TTT (1014F), TCG (1014S), and TGT (1014C) were not found. Accordingly, nine wild haplotypes, namely 1014L1, 1014L2, 1014L3, 1014L4, 1014L7, 1014L9, 1014L10, 1014L11, and 1014L21, respectively, were identified (Table 1), in the frequencies ranging from 1.12% (1/89) to 43.82% (39/89) in the three Laos populations (Fig. 2). In the LPY (Yot Ou County, Phongsaly Province) population, 1014L1 (46.99%, 39/83), 1014L4(9.64%, 8/83), 1014L7 (12.05%, 10/83), 1014L9 (7.23%, 6/83), and 1014L10 (19.28%, 16/83) had higher frequencies than other haplotypes. In the LCP (Pathoomphone County, Champasak Province) population, only 1014L3 was identified and accounted for 100% (4/4). In the LXP (Pak lay County, Xayabuli Province) population, 1014L3 and 1014L9 were both 50% (1/2). In addition, the newly identified susceptible haplotype, 1014L21, was uniquely distributed at LPY at a low frequency (1.20%, 1/83). Due to the time limit and different sample sizes between each sampling site, further evaluation is needed.

To analyze the genetic diversity indices and neutrality tests (Fu’s *Fs* and Tajima’s *D*) based on the *kdr* intron of *An. sinensis,* 89 *kdr* intron sequences in this study and 65 *kdr* intron sequences retrieved from the GenBank database (Additional file 3: Table S3a) were used in the subsequent analysis. A total of 14 haplotypes were found in nine populations. Genetic diversity varied significantly among geographical regions. The overall haplotype diversity (*Hd*) and nucleotide diversity (*Pi*) were 0.788 and 0.02190, respectively. Compared to the three *An. sinensis* populations in Laos, high haplotype diversity and nucleotide diversity were found in six populations from China. Moreover, significant departures from neutrality were detected by Fu’s *Fs* test in all the populations (−5.17700, *P* < 0.05), CN-GX (Guangxi Province, Southwest China) population (−4.51700, *P* < 0.02), and the CN-HaN (Hainan Province, Southwest China) population (−3.66700, *P* < 0.02), whereas they were not detected in all the Laos populations by Fu’s *Fs* or Tajima’s *D* test. Additionally, slightly insignificant departures from neutrality were detected by Fu’s *Fs* test in the CN-AH (Anhui Province, Central China) population (−1.50700,* P* < 0.1) and CN-YN (Yunnan Province, Southwest China) population (−1.19500,* P* < 0.1) (Additional file 3: Table S3a). Since the Fu’s *Fs* statistic is particularly sensitive to demographic effects, it is difficult to conclude whether positive selection or demographic history (e.g., population expansion) accounts for the observed pattern.

### Genealogical analysis of *kdr* mutations

**Fig. 3:**
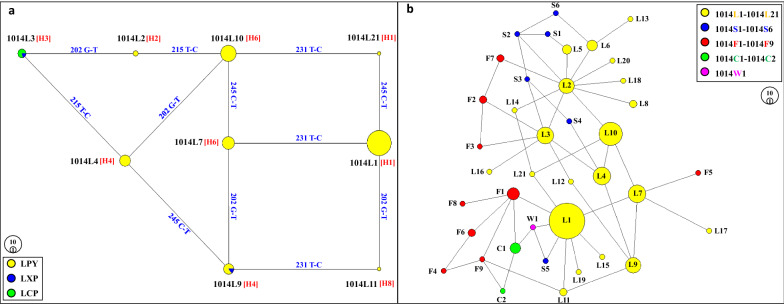
The network of *kdr* haplotypes identified in *An. sinensis* populations. **a** Networks showing the genealogical relationship in Laos. Yellow, green, and blue circles represent 1014L haplotypes from LPY (Phongsaly Province: Yot Ou County), LCP (Champasak Province: Pathoomphone County), and LXP (Xayabuli Province: Pak lay County) populations, respectively. The size of each circle is proportional to its corresponding frequencies. H represents the type of intron haplotypes (numbers in brackets). The straight line indicates the possible mutational step. The note above the line referred to the mutation position and base. **b** Networks showing the genealogical relationship among different *kdr* haplotypes from the present study and NCBI data. Yellow, blue, green, red, and pink circles represent 1014L, 1014S, 1014C, 1014F, and 1014 W haplotypes, respectively. The size of each circle is proportional to its corresponding frequencies

Network analysis showed that haplotypes H6-1014L7 and H8-1014L11 were derived from single mutational steps through T231C and G202T, respectively, in the intron 19 from ancestor H1-1014L1, while H1-1014L21 reported in this study derived from H1-1014L1 with a single mutation at C245T in the exon 20. Haplotype H3-1014L3, which was only detected in LXP and LCP populations, and H6-1014L10, were derived from single mutational steps through G202T and T215C, respectively, in the intron 19 from ancestor H2-1014L2. Additionally, H4-1014L4 was derived from H3-1014L3 with a single mutation at T215C in intron 19, while H4-1014L9 exhibited one more single mutation at C245T in exon 20 from the ancestor H3-1014L3 (Fig. 3a).

To estimate the evolutionary relationship of *kdr* mutations, nine haplotypes identified in this study and 30 haplotypes retrieved from the GenBank database (Table 1) were used to construct a network using Network 4.0. The network analysis revealed complex reticulate patterns and multiple independent mutation events leading to *kdr* haplotypes. The genealogical analysis revealed that a single mutation might result in the resistant phenotype from the susceptible one. For example, 1014S2 shared an identical intron sequence with 1014L2, L5, and S1, and a single mutation (T165C) might change the susceptible 1014L2 to the resistant 1014S2. Haplotypes 1014F1, F2, F3, F5, F7, and F9 were possibly derived from 1014L1, 1014L3, 1014L3, 1014L7, 1014L2, and 1014L11, respectively, while 1014F6 and F8 derived from the ancestor 1014L1 with two mutational steps, i.e., the additional mutations in exon 19 (T140A) or intron 19 (T229A) of 1014F1. Likewise, 1014F4 was derived from the ancestor 1014L11 with two mutational steps, i.e., the additional mutations in exon 19 (T140A) of 1014F9. Haplotypes 1014S1, S2, S3, S4, S5, and S6 were perhaps the results of an independent mutational step from six different wild haplotypes 1014L5, 1014L2, 1014L3, 1014L4, 1014L1, and 1014L6, respectively. Additionally, 1014W1 was derived from the ancestor 1014L1, while 1014C1 and 1014C2 were derived from 1014F1 with two or more mutational steps (Fig. 3b, Table 1).

### Mitochondrial DNA sequence variation

Eighty-nine sequences for *COII* were generated for the three populations (Additional file 3: Table S3b). The *COII* sequence alignment revealed 26 variable sites. The overall haplotype diversity (*Hd*), number of haplotypes, and nucleotide diversity (*Pi*) were 0.799, 22, and 0.00351, respectively. Significant departures from neutrality were both detected by Fu’s *Fs* test (−11.48900, *P* < 0.001) and Tajima’s *D* test (−1.79501, *P* < 0.02) in all the populations. In contrary to the LXP and LCP populations, high haplotype diversity (0.783), number of haplotypes (20), and nucleotide diversity (0.00282) were found in the LPY population. Additionally, significant departures from neutrality were both detected by Fu’s *Fs* test (−11.80300, *P* < 0.001) and Tajima’s *D* test (−1.61923, *P* < 0.05) in the LPY population, whereas not detected in the LXP and LCP population.

### Population structure and genetic differentiation

**Fig. 4:**
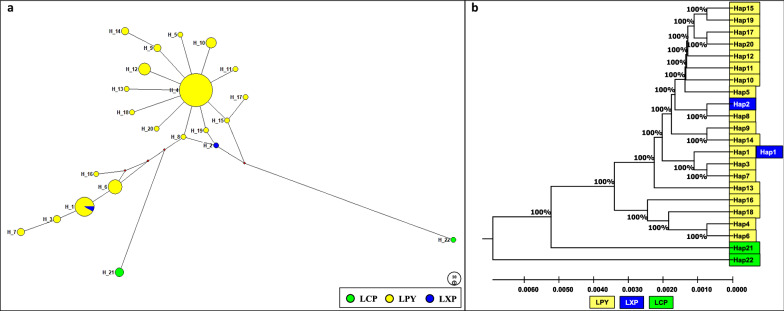
Phylogenetic analysis based on the *COII* sequences in *An. sinensis* populations in Laos. **a** Phylogenetic network of 22 mitochondrial haplotypes of the *COII* gene in *An. sinensis*. Localities are indicated by different colors (bottom right). The size of each circle is proportional to its corresponding frequencies. **b** UPGMA dendrogram based on Nei’s unbiased genetic distance between the 22 haplotypes of *An. sinensis*. Yellow, green, and blue circles/rectangles represent haplotypes from LPY (Phongsaly Province: Yot Ou County), LCP (Champasak Province: Pathoomphone County), and LXP (Xayabuli Province: Pak lay County) populations, respectively

The median-joining network based on 89 *COII* sequences denoted the distribution pattern exhibited by 22 haplotypes in *An. sinensis* populations. The *An. sinensis* populations fell into two main clusters. Cluster 1 consisted of all the haplotypes except for one haplotype (H22) from LCP; cluster 2 consisted of only one haplotype (H22). Within cluster 1, two sub-clusters were also found; sub-cluster 1 consisted of all the haplotypes in LPY and LXP, while sub-cluster 2 consisted of only one haplotype (H21) in LCP (Fig. 4a). The most common haplotypes referred to H1 (*n* = 13), H4 (*n* = 37), and H6 (*n* = 7), as only identified in 67.47% (56/83) of LPY and 50% (1/2) of LXP. H21 (*n* = 3) and H22 (*n* = 1) were only identified in LCP (Fig. 4a). The unweighted pair group method with arithmetic mean (UPGMA) dendrogram based on Nei’s unbiased genetic distances between haplotypes indicated that H22 constituted one cluster, while the other haplotypes constituted the second (Fig. 4b). Additionally, two sub-clusters were also found in the dendrogram, which was consistent with the results of the median-joining network.

To draw a broader comparison in haplotype from Laos and other geographical regions, we downloaded and analyzed available data in GenBank from neighboring nations (Additional file 2: Table S2). In general, a total of 148 *An. sinensis* *COII* sequences were generated for seven populations, including LPY (*n* = 83), LCP (*n* = 4), LXP (*n* = 2), JP (Japan, *n* = 3), KR (South Korea, *n* = 19), TH (Thailand, *n* = 3), and CN (China, *n* = 34), and 46 haplotypes were found in the median-joining network. The *An. sinensis* populations fell into two main clusters. Cluster 1 consisted of all the haplotypes except for one haplotype (H27) from LCP; cluster 2 consisted of only one haplotype (H27). Within cluster 1, three sub-clusters were also found: sub-cluster 1 consisted of haplotype (H26) from LCP; sub-cluster 2 consisted of five haplotypes (H29, H35, H37, H42, and H43) from China; sub-cluster 3 consisted of other haplotypes (Additional file 4: Fig. S1a). The UPGMA dendrogram based on Nei’s unbiased genetic distances between haplotypes indicated that H27 constituted one cluster, while the other haplotypes constituted the second (Additional file 4: Fig. S1b). Additionally, three sub-clusters were also found in the dendrogram, which was consistent with the results of the median-joining network.

AMOVA analysis based on *COII* sequences demonstrated that most of the variances were found among group variation (58.43%) rather than within populations (38.93%) and among populations within groups (2.64%), suggesting that these populations could fall into several groups. However, no statistical significance was found in evaluating the fixation index among groups (*F*_CT_), among populations within groups (*F*_SC_). In contrast, the fixation index within populations (*F*_ST_) showed statistical significance (*P* < 0.05) (Additional file 5: Table S4). Due to the time limit and different sample sizes between each sampling site, further evaluation is needed.

The maximal level of genetic differentiation by the fixation index *F*_ST_ based on sequences analysis of *COII* was between LCP and LPY (*F*_ST_ = 0.61657, *P *˂ 0.05). while no significant genetic differentiation was found between LPY and LXP populations (0.08915, *P* > 0.05) or between LXP and LCP populations (0.09565, *P* > 0.05). Furthermore, the minimal estimate of gene flow (*Nm*) was between LCP and LPY (*Nm* = 0.31093). In contrast, high gene flows were found between LPY and LXP populations (*Nm* = 5.10835), as well as between LXP and LCP populations (*Nm* = 4.72727) (Additional file 6: Table S5).

### Spatial genetic structure analysis, demographic history, and neutrality test

A Mantel test revealed a significant correlation between geographical and genetic distances in all populations (*Z* = 655.5437, *r* = 0.7995, *P* ≤ 0.0010), suggesting the genetic structure observed in *An. sinensis* populations could be partially explained by distance isolation based on *COII* sequence analysis (LCP and LPY populations) (Additional file 7: Fig. S2a). As indicated from Tajima’s *D* and Fu’s *Fs* tests based on *COII*, the LPY population exhibited significant negativity (*P* < 0.05, *P* < 0.001), suggesting a recent population expansion or selection (Table S3b). Furthermore, the observed smooth and unimodal mismatch distribution in the LPY population suggested a sudden population expansion, conforming to the mismatch distribution derived under the model of sudden expansion (Additional file 7: Fig. S2 b–d).

## Discussion

### The geographical isolation of *An. sinensis *populations from Lao PDR

Climate, society, and environmental factors can influence the spatial distribution of malaria vectors and disease transmission [51]. Geologically, 80% of the land in Laos is covered by mountains and plateaus, most of which are forested. The whole country is divided into Upper Laos, Central Laos, and Lower Laos from north to south. The terrain of Laos is high in the north and low in the south. In the north, Phongsaly Province borders China’s Yunnan-Guizhou Plateau, whereas Champasak Province borders Cambodia’s Stung Treng Province in the south, and the terrain is relatively flat. There was a strong association between *Anopheles* presence estimation and malaria prevalence. The malaria incidence is highest in southern Laos and lowest in the north, where malaria transmission is sporadic and local [3, 4].

A number of genetic markers were used to analyze population genomic data in *Anopheles*. mtDNA has been frequently used to resolve the issues of molecular taxonomy, phylogenetic relationships, and population structure in malaria vectors due to high copy numbers and the availability of conserved primers and PCR techniques [32]. In the case of recently diverged taxa or cryptic species of mosquitoes, however, ribosomal DNA (rDNA) has been shown to be more reliable than mtDNA in addressing evolutionary problems [52]. Microsatellites have been developed and used primarily to assess patterns and rates of gene flow among *Anopheles* populations due to their high mutation rate, while mtDNA polymorphisms are superior for detecting large-scale geographical differences [32]. In addition, for genotyping single biallelic single-nucleotide polymorphisms (SNPs) in large numbers of parasite or vector samples, the malaria genomics field has employed techniques such as Sequenom/Agena, TaqMan, and high-resolution melting curves. Nonetheless, SNP genotyping is inexpensive at scale but requires specialized instrumentation [53]. In the present study, significant genetic differentiation was identified between populations from the China–Laos border (LPY) and Cambodia–Laos border (LCP) by using mtDNA-COII sequencing (Fig. 4). The evident differences in the geographical distribution of *COII* allele between populations in the northern and the southern Laos are probably a result of the geographical barriers restricting gene flow, which was induced by the intricate landscape of Laos (Additional file 6: Table S5) (Additional file 7: Fig. S2a). In other words, spatial distance and heterogeneous landscape can be factors suppressing gene flow between the China–Laos border and Cambodia–Laos populations. The findings of distinct *An. sinensis* populations identified in Laos imply that local governments should undertake more targeted and effective malaria prevention and elimination measures.

### Effects of insecticide-associated selection

The intense utilization of insecticides for agricultural pest control and insecticide utilized in vector control might cause strong selective pressure for the resistance development in malaria vectors [54, 55]. Insecticide resistance occurs when the frequency of resistance genes in the mosquito population increases after exposure to insecticides [51]. Theoretically, selective pressure might exert an effect on shaping the frequency of insecticide resistance-related mutation in a population [30]. According to Souris’ study, the possibility of insecticide resistance was speculated by spatio-temporal models for *Anopheles* species’ presence, and was more remarkable in the southwest parts of the nation, particularly in the Champasak Province, owing to insecticide utilization [51]. However, in the present study, *kdr* mutations in the *vgsc* gene were not found in the Cambodia–Laos border (Champasak Province) (Fig. 2, Table 1). Nevertheless, it was improbable to evaluate the effect of a regional selective process of insecticides on the existing *kdr* distribution pattern, as the history of insecticide utilization was unknown for the specimens discussed herein.

Previous studies revealed that in the widespread species *An. sinensis,* the L1014F/L1014S/L1014C/L1014W/N1013S *kdr* mutations were identified in China [24, 25, 29, 56], while L1014F/L1014C were identified in Korea [28]. However, few studies focus on *kdr* mutations of *An. sinensis* in Laos. According to Verhaeghen’s study, *kdr* of *An. sinensis* (L1014S) was only found in southern Vietnam and in Cambodia near the Vietnamese border, but not in Laos [18]. Likewise, the present study demonstrates that only a wild haplotype was detected, TTG (1014L), which suggested that a further susceptibility bioassay or research on other common polymorphisms were needed to investigate the actual status of pyrethroid-based chemical insecticide resistance in Laos, especially the southwest part of the country. Intriguingly, there was geographical heterogeneity of wild *kdr* haplotypes and the presence of region-specific haplotypes (Fig. 3a, Table 1). For instance, 1014L3 was only identified along the Thailand–Laos border and Cambodia–Laos border. The newly identified haplotype, 1014L21, was only discovered on the China–Laos border and has not been identified in other countries (Fig. 3a, Table 1) [30, 57]. The region-specific distributions of *kdr* haplotypes highlight the necessity to continue the monitoring of chemical insecticide susceptibility to promptly detect potential occurrence and/or migration of chemical insecticide resistance in malaria vectors in Lao PDR.

Furthermore, previous studies revealed that the geographical distribution pattern and the genealogical analysis of *kdr* haplotypes strongly suggest that *kdr* variants have multiple origins [30, 31, 57]. Likewise, in the present study, we found the same pattern of the evolutionary relationship of 1014S and 1014F haplotypes when conducting network analysis combined with haplotypes retrieved from the GenBank (Fig. 3b). Interestingly, four newly identified *kdr* haplotypes were observed, namely 1014F7, 1014F8, 1014F9, and 1014W1. Haplotypes 1014F8 and 1014F9 were only identified in Zhejiang Province (Southeast China) and Anhui Province (Central China), while 1014W1 was only identified in Henan Province (Central China). The distinct distribution patterns of the 1014F/W allele are probably a result of independent mutation events in diverse geographical regions (Fig. 3b, Table1).

In addition, application of genetic hitchhiking by using neutrality tests revealed that the significant negative Fu’s *Fs* value in two southwest China populations (Guangxi and Hainan Provinces) could reflect the impact of intense selective pressure by the increased utilization of chemical insecticide on the *kdr* locus and the flanking regions of *kdr*, such as the *kdr* intron. In contrast, neutrality tests failed to provide evidence of selection at the *kdr* intron in Laos. The lack of strong selection pressure might partially elucidate the absence of chemical insecticide-resistant alleles in Laos (Additional file 3: Table S3a).

### History of insecticide-associated selection in Laos

Knowledge of insecticide resistance in *Anopheles* species is imperative for guiding malaria vector control strategies [58]. Exposing malaria vectors to insecticides is possibly predominantly driven by agricultural insecticides because of the intense agricultural insecticide input in contrast to public health insecticides [51]. However, the risk of insecticide resistance in malaria vectors in Southeast Asia is an underlying challenge for preventing malaria and the achievements seen during recent years [51]. Insecticide resistance in major malaria vectors has been identified in neighboring countries like Cambodia, Vietnam, and Thailand [55, 59], and the National Malaria Control Program has to determine potential regions of insecticide resistance emergence.

In Lao PDR, after the ban of DDT and the termination of the mass drug administration (MDA) program, ITNs and LLINs were introduced in 2000 [51]. Since then, vector control has relied on IRS and/or ITNs with the use of pyrethroids in this country [58]. According to the World Health Organization (WHO), approximately 50,403 LLINs were distributed to societies in 2018, accounting for a net coverage of 20% [4]. The greatest risk for insecticide resistance development is in the southwest part of Lao PDR; to be specific, rice and other agricultural systems along the Mekong River and the rest of the agricultural regions in Champasak [51]. However, previous limited studies revealed that no resistance to pyrethroids was found in several primary vectors including *An. maculatus*, *An. minimus*, and *An. sinensis* in Laos [18, 57], indicating that those insecticides remained suitable for malaria vector control. Likewise, in the present study, the lack of *kdr* mutations in *An. sinensis* samples from Laos is probably a result of the lack of intense local selection in combination with the geographical isolation in the mosquito populations. Since no regularly conducted vector control programs have been executed in the remote villages studied in Laos, the absence of pyrethroid selection pressure on the mosquito populations might explain why *kdr* is rare (Table 1).

Based on the findings in this study, we suggest that pyrethroids are still appropriate for controlling *An. sinensis* in Laos, and LLINs or IRS using pyrethroids remain valid for protecting people from indoor *Anopheles* bites and ought to be used extensively, particularly in the southern part of the country where malaria is endemic. A potent management program of chemical insecticide resistance should be executed to maintain the susceptibility of *vgsc* alleles. Furthermore, the application of pyrethroids should not be the only measure for malaria vector control and should be used in combination with insecticides with alternative modes of action or in a rotational manner [57]. As the precise quantity regarding insecticide usage in Lao PDR is unclear, a national registration system for insecticides should be established to guarantee the smooth monitoring of insecticides [51]. On the other hand, our findings can aid local governments in implementing targeted and effective vector control strategies for malaria prevention and elimination among the most vulnerable populations. Local chemical insecticide resistance surveillance systems in *Anopheles* mosquito populations should be implemented focusing on more restricted areas.

## Conclusions

The present study compares the genetic patterns revealed by a functional gene, *kdr*, with a neutral marker, *COII*, and demonstrates the combined impact of demographic and selection factors on the *An. sinensis* population structure. The strong genetic differentiation and limited gene flow between the China–Laos (northern Laos) and Cambodia–Laos (southern Laos) *An. sinensis* populations based on *COII* analysis suggest that these two regions are genetically isolated. Only nine wild haplotypes of *kdr*, TTG (1014L) were observed in *An. sinensis* populations, as well as distinct distribution of *kdr* wild haplotypes among different geological regions in Laos.

Lack of *kdr* mutations in the *vgsc* gene in the three border regions of Laos is likely a consequence of genetic isolation and the absence of intense selection pressure. The present study suggests that pyrethroids remain suitable for use against *An. sinensis* in Laos, but routine monitoring of chemical insecticide resistance should be continued and focused on more restricted areas in this country as part of chemical insecticide resistance management.

## Supplementary Information

**Additional file 1: Table S1.** Full list of 89 *An. sinensis* specimens collected in China–Laos, Thailand–Laos, and Cambodia–Laos borders. N/A, not identified. LXP, Pak lay County of Xayabuli Province (Thailand–Laos border); LPY, Yot Ou County of Phongsaly Province (China–Laos border); LCP, Pathoomphone County of Champasak Province (Cambodia–Laos border).

**Additional file 2: Table S2.** *COII* sequences of *An. sinensis* were downloaded from the NCBI. KR-YC, Yeoncheon (South Korea); KR-IC, Incheon (South Korea); KR-GR, Guryongpo (South Korea); CN-YN, Yunnan (China); Yunnan; CN-CQ, Chongqing (China); CN-AH, Anhui (China); TH-CM, Chiang Mai (Thailand); JP-NS, Nagasaki (Japan). N/A, no data.

**Additional file 3: Table S3. a** Genetic diversity indices and neutrality tests (Fu’s *Fs* and Tajima’s *D*) based on the *kdr* intron of *An. sinensis*. **b** Genetic diversity indices and neutrality tests (Fu’s *Fs* and Tajima’s *D*) based on the *COII* gene of *An. sinensis.* *nd* not determined; ^#^*P* < 0.10; *P < 0.05; **P < 0.02; ***P < 0.001. *n* number of sequences; *s* number of polymorphic sites; *pi* nucleotide diversity; *h* number of haplotypes; *Hd* haplotype diversity; *LPY* Yot Ou County (Phongsaly Province); *LXP* Pak lay County (Xayabuli Province); *LCP* Pathoomphone County (Champasak Province); *CN-AH* Anhui Province (China); *CN-GX* Guangxi Province (China); *CN-YN* Yunnan Province (China); *CN-HaN* Hainan Province (China); *CN-HeN* Henan Province (China); *CN-ZJ* Zhejiang Province (China).

**Additional file 4: Figure S1.** Phylogenetic analysis based on the *COII* sequences in *An. sinensis* populations from Laos and other countries. **a** Phylogenetic network of 46 mitochondrial haplotypes of the *COII* gene in *An. sinensis*. Localities are indicated by different colors (bottom right). The size of each circle is proportional to its corresponding frequencies. **b** UPGMA dendrogram based on Nei’s unbiased genetic distance between the 46 haplotypes of *An. sinensis*. Yellow, green, and blue circles/rectangles represent haplotypes found in this study from LPY (Phongsaly Province: Yot Ou County), LCP (Champasak Province: Pathoomphone County), and LXP (Xayabuli Province: Pak lay County) populations, respectively. Black, white, red, and sky blue circles/rectangles represent haplotypes found in NCBI data from Korea, Japan, China, and Thailand (Tables S2), respectively.

**Additional file 5: Table S4.** Analysis of molecular variance (AMOVA) of 10 *An. sinensis* populations based on *COII*. *F*_CT_, fixation index among groups; *F*_SC_, among populations within groups; *F*_ST_, within populations.

**Additional file 6: Table S5.** Genetic differentiation and gene flow among the geographical groups of *An. sinensis* based on *COII*. The pairwise *F*_ST_ values and *Nm* values based on the *COII* are shown below and above the diagonal, respectively. Characters in bold indicated the significance (*P* < 0.05). **Inf**, infinite. *LPY*, Yot Ou County, Phongsaly Province; *LXP*, Pak lay County, Xayabuli Province; *LCP*, Pathoomphone County, Champasak Province.

**Additional file 7: Figure S2. a** Isolation by distance; the relationship between geographical and genetic distances based on *COII* sequences in *An. sinensis* populations. Isolation by distance (IBD) was examined using a nonparametric Mantel test with the web-based computer program IBDWS v.3.16. **b**–**d** Mismatch distribution graphs for *An. sinensis* population based on *COII* sequences. The *x*- and *y*-axis show the number of pairwise differences and the frequency of the pairwise comparisons, respectively. The observed frequencies are represented by a dotted line. The frequency expected under the hypothesis of the constant population model is depicted by a solid line. *LPY*, Yot Ou County, Phongsaly Province; *LXP*, Pak lay County, Xayabuli Province; *LCP*, Pathoomphone County, Champasak Province.

## Data Availability

Data supporting the conclusions of this article are included within the article and its additional files. The current study’s datasets generated and/or analyzed are available in GenBank (http://www.ncbi.nlm.nih.gov/). The nucleotide sequence data reported in this study have been deposited in the GenBank DNA database (https://www.ncbi.nlm.nih.gov/genbank/) with accession number ON400573-ON400661.

## Abbreviations

GMS: Greater Mekong Subregion
Lao PDR: Lao People’s Democratic Republic
LLIN: Long-lasting insecticide-treated nets
IRS: Indoor residual spraying
VGSC: Voltage-gated sodium channel protein
mtDNA: Mitochondrial DNA
*COII*: Cytochrome c oxidase subunit II
