# Supplementary material for: Population genetic structure and evolutionary genetics of Anopheles sinensis based on knockdown resistance (kdr) mutations and mtDNA-COII gene in China–Laos, Thailand–Laos, and Cambodia–Laos borders
Source: Parasit Vectors. 2022 Jun 26;15:229. doi: 10.1186/s13071-022-05366-9 (PMC9233850; doi:10.1186/s13071-022-05366-9)
Supplement: Supplementary file 1 — Additional file 1: Table S1. Full list of 89 An. sinensis specimens collected in China–Laos, Thailand–Laos, and Cambodia–Laos borders. N/A, not identified. LXP, Pak lay County of Xayabuli Province (Thailand–Laos border); LPY, Yot Ou County of Phongsaly Province (China–Laos border); LCP, Pathoomphone County of Champasak Province (Cambodia–Laos border). [file 13071_2022_5366_MOESM1_ESM.docx]

**Tables S1. Full list of 89 *Anopheles sinensis* specimens collected in China-Laos, Thailand-Laos, and Cambodia-Laos borders^a^.**

| **NO.** | **ID** | **Morphology species ID** | **Molecular species ID based on ITS2** | **Molecular species ID based on COII** | **Species** | **Location** | **Latitude** | **Longitude** |
| --- | --- | --- | --- | --- | --- | --- | --- | --- |
| 1 | LXP1 | *An. sinensis* | *An. sinensis LXP1* | *An. sinensis LXP1* | *An. sinensis* | Xayabuli Province: Pak lay County | 101°82' | 19°39' |
| 2 | LXP2 | *An. sinensis* | N/A | *An. sinensis LXP2* | N/A | Xayabuli Province: Pak lay County | 101°82' | 19°39' |
| 3 | LXP4 | *An. sinensis* | *An. sinensis LXP4* | *An. sinensis LXP4* | *An. sinensis* | Xayabuli Province: Pak lay County | 101°82' | 19°39' |
| 4 | LPY1 | *An. sinensis* | *An. sinensis LPY1* | N/A | N/A | Xayabuli Province: Pak lay County | 101°82' | 19°39' |
| 5 | LPY2 | *An. sinensis* | *An. sinensis LPY2* | *An. sinensis LPY2* | *An. sinensis* | Phongsaly Province: Yot Ou County | 101°79' | 22°12' |
| 6 | LPY3 | *An. sinensis* | *An. sinensis LPY3* | N/A | N/A | Phongsaly Province: Yot Ou County | 101°79' | 22°12' |
| 7 | LPY4 | *An. sinensis* | *An. sinensis LPY4* | *An. sinensis LPY4* | *An. sinensis* | Phongsaly Province: Yot Ou County | 101°79' | 22°12' |
| 8 | LPY5 | *An. sinensis* | *An. sinensis LPY5* | N/A | N/A | Phongsaly Province: Yot Ou County | 101°79' | 22°12' |
| 9 | LPY6 | *An. sinensis* | *An. sinensis LPY6* | N/A | N/A | Phongsaly Province: Yot Ou County | 101°79' | 22°12' |
| 10 | LPY7 | *An. sinensis* | *An. sinensis LPY7* | N/A | N/A | Phongsaly Province: Yot Ou County | 101°79' | 22°12' |
| 11 | LPY8 | *An. sinensis* | *An. sinensis LPY8* | *An. sinensis LPY8* | *An. sinensis* | Phongsaly Province: Yot Ou County | 101°79' | 22°12' |
| 12 | LPY9 | *An. sinensis* | *An. sinensis LPY9* | *An. sinensis LPY9* | *An. sinensis* | Phongsaly Province: Yot Ou County | 101°79' | 22°12' |
| 13 | LPY10 | *An. sinensis* | *An. sinensis LPY10* | *An. sinensis LPY10* | *An. sinensis* | Phongsaly Province: Yot Ou County | 101°79' | 22°12' |
| 14 | LPY11 | *An. sinensis* | *An. sinensis LPY11* | *An. sinensis LPY11* | *An. sinensis* | Phongsaly Province: Yot Ou County | 101°79' | 22°12' |
| 15 | LPY12 | *An. sinensis* | N/A | *An. sinensis LPY12* | N/A | Phongsaly Province: Yot Ou County | 101°79' | 22°12' |
| 16 | LPY13 | *An. sinensis* | *An. sinensis LPY13* | *An. sinensis LPY13* | *An. sinensis* | Phongsaly Province: Yot Ou County | 101°79' | 22°12' |
| 17 | LPY14 | *An. sinensis* | *An. sinensis LPY14* | *An. sinensis LPY14* | *An. sinensis* | Phongsaly Province: Yot Ou County | 101°79' | 22°12' |
| 18 | LPY15 | *An. sinensis* | *An. sinensis LPY15* | *An. sinensis LPY15* | *An. sinensis* | Phongsaly Province: Yot Ou County | 101°79' | 22°12' |
| 19 | LPY16 | *An. sinensis* | *An. sinensis LPY16* | *An. sinensis LPY16* | *An. sinensis* | Phongsaly Province: Yot Ou County | 101°79' | 22°12' |
| 20 | LPY17 | *An. sinensis* | *An. sinensis LPY17* | *An. sinensis LPY17* | *An. sinensis* | Phongsaly Province: Yot Ou County | 101°79' | 22°12' |
| 21 | LPY18 | *An. sinensis* | N/A | *An. sinensis LPY18* | N/A | Phongsaly Province: Yot Ou County | 101°79' | 22°12' |
| 22 | LPY19 | *An. sinensis* | *An. sinensis LPY19* | *An. sinensis LPY19* | *An. sinensis* | Phongsaly Province: Yot Ou County | 101°79' | 22°12' |
| 23 | LPY20 | *An. sinensis* | *An. sinensis LPY20* | N/A | N/A | Phongsaly Province: Yot Ou County | 101°79' | 22°12' |
| 24 | LPY21 | *An. sinensis* | *An. sinensis LPY21* | *An. sinensis LPY21* | *An. sinensis* | Phongsaly Province: Yot Ou County | 101°79' | 22°12' |
| 25 | LPY22 | *An. sinensis* | *An. sinensis LPY22* | *An. sinensis LPY22* | *An. sinensis* | Phongsaly Province: Yot Ou County | 101°79' | 22°12' |
| 26 | LPY23 | *An. sinensis* | *An. sinensis LPY23* | *An. sinensis LPY23* | *An. sinensis* | Phongsaly Province: Yot Ou County | 101°79' | 22°12' |
| 27 | LPY24 | *An. sinensis* | N/A | *An. sinensis LPY24* | N/A | Phongsaly Province: Yot Ou County | 101°79' | 22°12' |
| 28 | LPY25 | *An. sinensis* | *An. sinensis LPY25* | *An. sinensis LPY25* | *An. sinensis* | Phongsaly Province: Yot Ou County | 101°79' | 22°12' |
| 29 | LPY26 | *An. sinensis* | *An. sinensis LPY26* | *An. sinensis LPY26* | *An. sinensis* | Phongsaly Province: Yot Ou County | 101°79' | 22°12' |
| 30 | LPY27 | *An. sinensis* | *An. sinensis LPY27* | N/A | N/A | Phongsaly Province: Yot Ou County | 101°79' | 22°12' |
| 31 | LPY29 | *An. sinensis* | *An. sinensis LPY29* | *An. sinensis LPY29* | *An. sinensis* | Phongsaly Province: Yot Ou County | 101°79' | 22°12' |
| 32 | LPY30 | *An. sinensis* | *An. sinensis LPY30* | *An. sinensis LPY30* | *An. sinensis* | Phongsaly Province: Yot Ou County | 101°79' | 22°12' |
| 33 | LPY31 | *An. sinensis* | *An. sinensis LPY31* | *An. sinensis LPY31* | *An. sinensis* | Phongsaly Province: Yot Ou County | 101°79' | 22°12' |
| 34 | LPY32 | *An. sinensis* | N/A | *An. sinensis LPY32* | *An. sinensis* | Phongsaly Province: Yot Ou County | 101°79' | 22°12' |
| 35 | LPY33 | *An. sinensis* | *An. sinensis LPY33* | *An. sinensis LPY33* | *An. sinensis* | Phongsaly Province: Yot Ou County | 101°79' | 22°12' |
| 36 | LPY34 | *An. sinensis* | *An. sinensis LPY34* | N/A | N/A | Phongsaly Province: Yot Ou County | 101°79' | 22°12' |
| 37 | LPY35 | *An. sinensis* | *An. sinensis LPY35* | *An. sinensis LPY35* | *An. sinensis* | Phongsaly Province: Yot Ou County | 101°79' | 22°12' |
| 38 | LPY36 | *An. sinensis* | *An. sinensis LPY36* | *An. sinensis LPY36* | *An. sinensis* | Phongsaly Province: Yot Ou County | 101°79' | 22°12' |
| 39 | LPY37 | *An. sinensis* | *An. sinensis LPY37* | *An. sinensis LPY37* | *An. sinensis* | Phongsaly Province: Yot Ou County | 101°79' | 22°12' |
| 40 | LPY38 | *An. sinensis* | *An. sinensis LPY38* | *An. sinensis LPY38* | *An. sinensis* | Phongsaly Province: Yot Ou County | 101°79' | 22°12' |
| 41 | LPY39 | *An. sinensis* | *An. sinensis LPY39* | *An. sinensis LPY39* | *An. sinensis* | Phongsaly Province: Yot Ou County | 101°79' | 22°12' |
| 42 | LPY40 | *An. sinensis* | N/A | *An. sinensis LPY40* | N/A | Phongsaly Province: Yot Ou County | 101°79' | 22°12' |
| 43 | LPY41 | *An. sinensis* | *An. sinensis LPY41* | *An. sinensis LPY41* | *An. sinensis* | Phongsaly Province: Yot Ou County | 101°79' | 22°12' |
| 44 | LPY42 | *An. sinensis* | N/A | *An. sinensis LPY42* | N/A | Phongsaly Province: Yot Ou County | 101°79' | 22°12' |
| 45 | LPY43 | *An. sinensis* | *An. sinensis LPY43* | *An. sinensis LPY43* | *An. sinensis* | Phongsaly Province: Yot Ou County | 101°79' | 22°12' |
| 46 | LPY44 | *An. sinensis* | *An. sinensis LPY44* | *An. sinensis LPY44* | *An. sinensis* | Phongsaly Province: Yot Ou County | 101°79' | 22°12' |
| 47 | LPY45 | *An. sinensis* | *An. sinensis LPY45* | *An. sinensis LPY45* | *An. sinensis* | Phongsaly Province: Yot Ou County | 101°79' | 22°12' |
| 48 | LPY46 | *An. sinensis* | *An. sinensis LPY46* | *An. sinensis LPY46* | *An. sinensis* | Phongsaly Province: Yot Ou County | 101°79' | 22°12' |
| 49 | LPY47 | *An. sinensis* | *An. sinensis LPY47* | *An. sinensis LPY47* | *An. sinensis* | Phongsaly Province: Yot Ou County | 101°79' | 22°12' |
| 50 | LPY48 | *An. sinensis* | *An. sinensis LPY48* | *An. sinensis LPY48* | *An. sinensis* | Phongsaly Province: Yot Ou County | 101°79' | 22°12' |
| 51 | LPY49 | *An. sinensis* | *An. sinensis LPY49* | *An. sinensis LPY49* | *An. sinensis* | Phongsaly Province: Yot Ou County | 101°79' | 22°12' |
| 52 | LPY50 | *An. sinensis* | *An. sinensis LPY50* | *An. sinensis LPY50* | *An. sinensis* | Phongsaly Province: Yot Ou County | 101°79' | 22°12' |
| 53 | LPY51 | *An. sinensis* | *An. sinensis LPY51* | *An. sinensis LPY51* | *An. sinensis* | Phongsaly Province: Yot Ou County | 101°79' | 22°12' |
| 54 | LPY52 | *An. sinensis* | *An. sinensis LPY52* | *An. sinensis LPY52* | *An. sinensis* | Phongsaly Province: Yot Ou County | 101°79' | 22°12' |
| 55 | LPY53 | *An. sinensis* | *An. sinensis LPY53* | *An. sinensis LPY53* | *An. sinensis* | Phongsaly Province: Yot Ou County | 101°79' | 22°12' |
| 56 | LPY54 | *An. sinensis* | *An. sinensis LPY54* | *An. sinensis LPY54* | *An. sinensis* | Phongsaly Province: Yot Ou County | 101°79' | 22°12' |
| 57 | LPY55 | *An. sinensis* | *An. sinensis LPY55* | N/A | N/A | Phongsaly Province: Yot Ou County | 101°79' | 22°12' |
| 58 | LPY56 | *An. sinensis* | *An. sinensis LPY56* | *An. sinensis LPY56* | *An. sinensis* | Phongsaly Province: Yot Ou County | 101°79' | 22°12' |
| 59 | LPY57 | *An. sinensis* | *An. sinensis LPY57* | *An. sinensis LPY57* | *An. sinensis* | Phongsaly Province: Yot Ou County | 101°79' | 22°12' |
| 60 | LPY58 | *An. sinensis* | N/A | *An. sinensis LPY58* | N/A | Phongsaly Province: Yot Ou County | 101°79' | 22°12' |
| 61 | LPY59 | *An. sinensis* | *An. sinensis LPY59* | *An. sinensis LPY59* | *An. sinensis* | Phongsaly Province: Yot Ou County | 101°79' | 22°12' |
| 62 | LPY60 | *An. sinensis* | *An. sinensis LPY60* | *An. sinensis LPY60* | *An. sinensis* | Phongsaly Province: Yot Ou County | 101°79' | 22°12' |
| 63 | LPY61 | *An. sinensis* | *An. sinensis LPY61* | *An. sinensis LPY61* | *An. sinensis* | Phongsaly Province: Yot Ou County | 101°79' | 22°12' |
| 64 | LPY62 | *An. sinensis* | *An. sinensis LPY62* | *An. sinensis LPY62* | *An. sinensis* | Phongsaly Province: Yot Ou County | 101°79' | 22°12' |
| 65 | LPY63 | *An. sinensis* | *An. sinensis LPY63* | *An. sinensis LPY63* | *An. sinensis* | Phongsaly Province: Yot Ou County | 101°79' | 22°12' |
| 66 | LPY64 | *An. sinensis* | *An. sinensis LPY64* | *An. sinensis LPY64* | *An. sinensis* | Phongsaly Province: Yot Ou County | 101°79' | 22°12' |
| 67 | LPY65 | *An. sinensis* | *An. sinensis LPY65* | *An. sinensis LPY65* | *An. sinensis* | Phongsaly Province: Yot Ou County | 101°79' | 22°12' |
| 68 | LPY66 | *An. sinensis* | *An. sinensis LPY66* | *An. sinensis LPY66* | *An. sinensis* | Phongsaly Province: Yot Ou County | 101°79' | 22°12' |
| 69 | LPY67 | *An. sinensis* | *An. sinensis LPY67* | *An. sinensis LPY67* | *An. sinensis* | Phongsaly Province: Yot Ou County | 101°79' | 22°12' |
| 70 | LPY68 | *An. sinensis* | *An. sinensis LPY68* | N/A | N/A | Phongsaly Province: Yot Ou County | 101°79' | 22°12' |
| 71 | LPY69 | *An. sinensis* | *An. sinensis LPY69* | *An. sinensis LPY69* | *An. sinensis* | Phongsaly Province: Yot Ou County | 101°79' | 22°12' |
| 72 | LPY70 | *An. sinensis* | *An. sinensis LPY70* | *An. sinensis LPY70* | *An. sinensis* | Phongsaly Province: Yot Ou County | 101°79' | 22°12' |
| 73 | LPY75 | *An. sinensis* | *An. sinensis LPY75* | N/A | N/A | Phongsaly Province: Yot Ou County | 101°79' | 22°12' |
| 74 | LPY77 | *An. sinensis* | *An. sinensis LPY77* | N/A | N/A | Phongsaly Province: Yot Ou County | 101°79' | 22°12' |
| 75 | LPY78 | *An. sinensis* | *An. sinensis LPY78* | N/A | N/A | Phongsaly Province: Yot Ou County | 101°79' | 22°12' |
| 76 | LPY79 | *An. sinensis* | *An. sinensis LPY79* | *An. sinensis LPY79* | *An. sinensis* | Phongsaly Province: Yot Ou County | 101°79' | 22°12' |
| 77 | LPY80 | *An. sinensis* | *An. sinensis LPY80* | *An. sinensis LPY80* | *An. sinensis* | Phongsaly Province: Yot Ou County | 101°79' | 22°12' |
| 78 | LPY81 | *An. sinensis* | *An. sinensis LPY81* | *An. sinensis LPY81* | *An. sinensis* | Phongsaly Province: Yot Ou County | 101°79' | 22°12' |
| 79 | LPY82 | *An. sinensis* | *An. sinensis LPY82* | *An. sinensis LPY82* | *An. sinensis* | Phongsaly Province: Yot Ou County | 101°79' | 22°12' |
| 80 | LPY83 | *An. sinensis* | *An. sinensis LPY83* | *An. sinensis LPY83* | *An. sinensis* | Phongsaly Province: Yot Ou County | 101°79' | 22°12' |
| 81 | LPY84 | *An. sinensis* | *An. sinensis LPY84* | *An. sinensis LPY84* | *An. sinensis* | Phongsaly Province: Yot Ou County | 101°79' | 22°12' |
| 82 | LPY85 | *An. sinensis* | *An. sinensis LPY85* | *An. sinensis LPY85* | *An. sinensis* | Phongsaly Province: Yot Ou County | 101°79' | 22°12' |
| 83 | LPY86 | *An. sinensis* | *An. sinensis LPY86* | *An. sinensis LPY86* | *An. sinensis* | Phongsaly Province: Yot Ou County | 101°79' | 22°12' |
| 84 | LPY87 | *An. sinensis* | *An. sinensis LPY87* | *An. sinensis LPY87* | *An. sinensis* | Phongsaly Province: Yot Ou County | 101°79' | 22°12' |
| 85 | LPY88 | *An. sinensis* | *An. sinensis LPY88* | N/A | N/A | Phongsaly Province: Yot Ou County | 101°79' | 22°12' |
| 86 | LPY89 | *An. sinensis* | *An. sinensis LPY89* | *An. sinensis LPY89* | *An. sinensis* | Phongsaly Province: Yot Ou County | 101°79' | 22°12' |
| 87 | LPY90 | *An. sinensis* | *An. sinensis LPY90* | *An. sinensis LPY90* | *An. sinensis* | Phongsaly Province: Yot Ou County | 101°79' | 22°12' |
| 88 | LPY91 | *An. sinensis* | *An. sinensis LPY91* | *An. sinensis LPY91* | *An. sinensis* | Phongsaly Province: Yot Ou County | 101°79' | 22°12' |
| 89 | LPY92 | *An. sinensis* | N/A | *An. sinensis LPY92* | N/A | Phongsaly Province: Yot Ou County | 101°79' | 22°12' |
| 90 | LPY93 | *An. sinensis* | *An. sinensis LPY93* | *An. sinensis LPY93* | *An. sinensis* | Phongsaly Province: Yot Ou County | 101°79' | 22°12' |
| 91 | LPY94 | *An. sinensis* | *An. sinensis LPY94* | *An. sinensis LPY94* | *An. sinensis* | Phongsaly Province: Yot Ou County | 101°79' | 22°12' |
| 92 | LPY95 | *An. sinensis* | *An. sinensis LPY95* | *An. sinensis LPY95* | *An. sinensis* | Phongsaly Province: Yot Ou County | 101°79' | 22°12' |
| 93 | LPY96 | *An. sinensis* | *An. sinensis LPY96* | *An. sinensis LPY96* | *An. sinensis* | Phongsaly Province: Yot Ou County | 101°79' | 22°12' |
| 94 | LPY97 | *An. sinensis* | *An. sinensis LPY97* | *An. sinensis LPY97* | *An. sinensis* | Phongsaly Province: Yot Ou County | 101°79' | 22°12' |
| 95 | LPY98 | *An. sinensis* | *An. sinensis LPY98* | N/A | N/A | Phongsaly Province: Yot Ou County | 101°79' | 22°12' |
| 96 | LPY99 | *An. sinensis* | *An. sinensis LPY99* | N/A | N/A | Phongsaly Province: Yot Ou County | 101°79' | 22°12' |
| 97 | LPY100 | *An. sinensis* | *An. sinensis LPY100* | N/A | N/A | Phongsaly Province: Yot Ou County | 101°79' | 22°12' |
| 98 | LPY102 | *An. sinensis* | *An. sinensis LPY102* | N/A | N/A | Phongsaly Province: Yot Ou County | 101°79' | 22°12' |
| 99 | LPY103 | *An. sinensis* | *An. sinensis LPY103* | N/A | N/A | Phongsaly Province: Yot Ou County | 101°79' | 22°12' |
| 100 | LPY104 | *An. sinensis* | *An. sinensis LPY104* | N/A | N/A | Phongsaly Province: Yot Ou County | 101°79' | 22°12' |
| 101 | LPY105 | *An. sinensis* | *An. sinensis LPY105* | N/A | N/A | Phongsaly Province: Yot Ou County | 101°79' | 22°12' |
| 102 | LPY107 | *An. sinensis* | *An. sinensis LPY107* | N/A | N/A | Phongsaly Province: Yot Ou County | 101°79' | 22°12' |
| 103 | LPY108 | *An. sinensis* | *An. sinensis LPY108* | N/A | N/A | Phongsaly Province: Yot Ou County | 101°79' | 22°12' |
| 104 | LPY109 | *An. sinensis* | *An. sinensis LPY109* | *An. sinensis LPY109* | *An. sinensis* | Phongsaly Province: Yot Ou County | 101°79' | 22°12' |
| 105 | LPY110 | *An. sinensis* | *An. sinensis LPY110* | N/A | N/A | Phongsaly Province: Yot Ou County | 101°79' | 22°12' |
| 106 | LPY111 | *An. sinensis* | N/A | *An. sinensis LPY111* | N/A | Phongsaly Province: Yot Ou County | 101°79' | 22°12' |
| 107 | LPY112 | *An. sinensis* | *An. sinensis LPY112* | N/A | N/A | Phongsaly Province: Yot Ou County | 101°79' | 22°12' |
| 108 | LPY113 | *An. sinensis* | *An. sinensis LPY113* | N/A | N/A | Phongsaly Province: Yot Ou County | 101°79' | 22°12' |
| 109 | LPY114 | *An. sinensis* | *An. sinensis LPY114* | N/A | N/A | Phongsaly Province: Yot Ou County | 101°79' | 22°12' |
| 110 | LPY115 | *An. sinensis* | *An. sinensis LPY115* | N/A | N/A | Phongsaly Province: Yot Ou County | 101°79' | 22°12' |
| 111 | LPY117 | *An. sinensis* | *An. sinensis LPY117* | N/A | N/A | Phongsaly Province: Yot Ou County | 101°79' | 22°12' |
| 112 | LPY118 | *An. sinensis* | *An. sinensis LPY118* | N/A | N/A | Phongsaly Province: Yot Ou County | 101°79' | 22°12' |
| 113 | LPY119 | *An. sinensis* | *An. sinensis LPY119* | N/A | N/A | Phongsaly Province: Yot Ou County | 101°79' | 22°12' |
| 114 | LPY120 | *An. sinensis* | *An. sinensis LPY120* | N/A | N/A | Phongsaly Province: Yot Ou County | 101°79' | 22°12' |
| 115 | LPY121 | *An. sinensis* | *An. sinensis LPY121* | *An. sinensis LPY121* | *An. sinensis* | Phongsaly Province: Yot Ou County | 101°79' | 22°12' |
| 116 | LPY122 | *An. sinensis* | *An. sinensis LPY122* | *An. sinensis LPY122* | *An. sinensis* | Phongsaly Province: Yot Ou County | 101°79' | 22°12' |
| 117 | LPY123 | *An. sinensis* | *An. sinensis LPY123* | *An. sinensis LPY123* | *An. sinensis* | Phongsaly Province: Yot Ou County | 101°79' | 22°12' |
| 118 | LPY124 | *An. sinensis* | *An. sinensis LPY124* | N/A | N/A | Phongsaly Province: Yot Ou County | 101°79' | 22°12' |
| 119 | LPY125 | *An. sinensis* | *An. sinensis LPY125* | *An. sinensis LPY125* | *An. sinensis* | Phongsaly Province: Yot Ou County | 101°79' | 22°12' |
| 120 | LPY126 | *An. sinensis* | *An. sinensis LPY126* | *An. sinensis LPY126* | *An. sinensis* | Phongsaly Province: Yot Ou County | 101°79' | 22°12' |
| 121 | LPY127 | *An. sinensis* | *An. sinensis LPY127* | *An. sinensis LPY127* | *An. sinensis* | Phongsaly Province: Yot Ou County | 101°79' | 22°12' |
| 122 | LPY128 | *An. sinensis* | *An. sinensis LPY128* | N/A | N/A | Phongsaly Province: Yot Ou County | 101°79' | 22°12' |
| 123 | LPY129 | *An. sinensis* | *An. sinensis LPY129* | *An. sinensis LPY129* | *An. sinensis* | Phongsaly Province: Yot Ou County | 101°79' | 22°12' |
| 124 | LPY130 | *An. sinensis* | *An. sinensis LPY130* | *An. sinensis LPY130* | *An. sinensis* | Phongsaly Province: Yot Ou County | 101°79' | 22°12' |
| 125 | LPY131 | *An. sinensis* | *An. sinensis LPY131* | *An. sinensis LPY131* | *An. sinensis* | Phongsaly Province: Yot Ou County | 101°79' | 22°12' |
| 126 | LPY132 | *An. sinensis* | *An. sinensis LPY132* | *An. sinensis LPY132* | *An. sinensis* | Phongsaly Province: Yot Ou County | 101°79' | 22°12' |
| 127 | LPY133 | *An. sinensis* | *An. sinensis LPY133* | *An. sinensis LPY133* | *An. sinensis* | Phongsaly Province: Yot Ou County | 101°79' | 22°12' |
| 128 | LPY134 | *An. sinensis* | *An. sinensis LPY134* | *An. sinensis LPY134* | *An. sinensis* | Phongsaly Province: Yot Ou County | 101°79' | 22°12' |
| 129 | LPY-Q6 | *An. tessellatus* | *An. sinensis LPY-Q6* | *An. sinensis LPY-Q6* | *An. sinensis* | Phongsaly Province: Yot Ou County | 101°79' | 22°12' |
| 130 | LCP25 | *An. sinensis* | *An. sinensis LCB25* | *An. sinensis LCB25* | *An. sinensis* | Champasak Province: Pathoomphone County | 106°04' | 14°43' |
| 131 | LCP47 | *An. sinensis* | *An. sinensis LCB47* | *An. sinensis LCB47* | *An. sinensis* | Champasak Province: Pathoomphone County | 106°04' | 14°43' |
| 132 | LCP55 | *An. sinensis* | *An. sinensis LCB55* | *An. sinensis LCB55* | *An. sinensis* | Champasak Province: Pathoomphone County | 106°04' | 14°43' |
| 133 | LCP73 | *An. sinensis* | *An. sinensis LCB73* | *An. sinensis LCB73* | *An. sinensis* | Champasak Province: Pathoomphone County | 106°04' | 14°43' |
| 134 | LCP36 | *An. sinensis* | *An. sinensis LCB36* | N/A | N/A | Champasak Province: Pathoomphone County | 106°04' | 14°43' |

a, data collected and analyzed in accordance with our previous study [33].

N/A, not identified. LXP, Pak lay County of Xayabuli Province (Thailand-Laos border); LPY, Yot Ou County of Phongsaly Province (China-Laos border); LCP, Pathoomphone County of Champasak Province (Cambodia-Laos border).
