# Supplementary material for: Population genetic structure and evolutionary genetics of Anopheles sinensis based on knockdown resistance (kdr) mutations and mtDNA-COII gene in China–Laos, Thailand–Laos, and Cambodia–Laos borders
Source: Parasit Vectors. 2022 Jun 26;15:229. doi: 10.1186/s13071-022-05366-9 (PMC9233850; doi:10.1186/s13071-022-05366-9)
Supplement: Supplementary file 2 — Additional file 2: Table S2. COII sequences of An. sinensis were downloaded from the NCBI. KR-YC, Yeoncheon (South Korea); KR-IC, Incheon (South Korea); KR-GR, Guryongpo (South Korea); CN-YN, Yunnan (China); Yunnan; CN-CQ, Chongqing (China); CN-AH, Anhui (China); TH-CM, Chiang Mai (Thailand); JP-NS, Nagasaki (Japan). N/A, no data. [file 13071_2022_5366_MOESM2_ESM.docx]

**Table S2. COII sequences of *Anopheles sinensis* downloaded from the NCBI**

| **Sequence ID** | **GenBank ID** | **Location** | **longitude** | **Latitude** | **Author** |
| --- | --- | --- | --- | --- | --- |
| KR-YC1 | KX840670.1 | South Korea, Yeoncheon | 127.100 | 38.133 | Kang, S., et al. |
| KR-YC2 | KX840669.1 | South Korea, Yeoncheon | 127.100 | 38.133 | Kang, S., et al. |
| KR-YC3 | KX840668.1 | South Korea, Yeoncheon | 127.100 | 38.133 | Kang, S., et al. |
| KR-YC4 | KX840667.1 | South Korea, Yeoncheon | 127.100 | 38.133 | Kang, S., et al. |
| KR-YC5 | KX840666.1 | South Korea, Yeoncheon | 127.100 | 38.133 | Kang, S., et al. |
| KR-YC6 | KX840665.1 | South Korea, Yeoncheon | 127.100 | 38.133 | Kang, S., et al. |
| KR-YC7 | KX840664.1 | South Korea, Yeoncheon | 127.100 | 38.133 | Kang, S., et al. |
| KR-YC8 | KX840663.1 | South Korea, Yeoncheon | 127.100 | 38.133 | Kang, S., et al. |
| KR-YC9 | KX840662.1 | South Korea, Yeoncheon | 127.100 | 38.133 | Kang, S., et al. |
| KR-YC10 | KX840661.1 | South Korea, Yeoncheon | 127.100 | 38.133 | Kang, S., et al. |
| KR-YC11 | KX840660.1 | South Korea, Yeoncheon | 127.100 | 38.133 | Kang, S., et al. |
| KR-YC12 | KX840659.1 | South Korea, Yeoncheon | 127.100 | 38.133 | Kang, S., et al. |
| KR-YC13 | KX840658.1 | South Korea, Yeoncheon | 127.100 | 38.133 | Kang, S., et al. |
| KR-YC14 | KX840653.1 | South Korea, Yeoncheon | 127.100 | 38.133 | Kang, S., et al. |
| KR-YC15 | KX840652.1 | South Korea, Yeoncheon | 127.100 | 38.133 | Kang, S., et al. |
| KR-GB1 | KX840655.1 | South Korea, Yeoncheon | 129.551 | 35.973 | Kang, S., et al. |
| KR-GB2 | KX840654.1 | South Korea, Yeoncheon | 129.551 | 35.973 | Kang, S., et al. |
| JP-NS1 | EU931617.1 | Japan, Nagasaki | 129.877 | 32.753 | Park,M.-H., et al. |
| JP-NS2 | EU931616.1 | Japan, Nagasaki | 129.877 | 32.753 | Park,M.-H., et al. |
| JP-NS3 | EU931615.1 | Japan, Nagasaki | 129.877 | 32.753 | Park,M.-H., et al. |
| TH-CM1 | AY130468.1 | Thailand, Chiang Mai, Maetang district | 98.941 | 19.105 | Min, G.-S., et al. |
| TH-CM2 | AY130467.1 | Thailand, Chiang Mai, Maetang district | 98.941 | 19.105 | Min, G.-S., et al. |
| TH-CM3 | AY130466.1 | Thailand, Chiang Mai, Maetang district | 98.941 | 19.105 | Min, G.-S., et al. |
| KR-IC1 | AY130465.1 | South Korea, Incheon | 126.647 | 37.466 | Min, G.-S., et al. |
| KR-IC2 | AY130464.1 | South Korea, Incheon | 126.647 | 37.466 | Min, G.-S., et al. |
| CN-YN1 | MG816568.1 | China, Yunnan | N/A | N/A | Ding,Y.-R., et al. |
| CN-YN2 | MG816567.1 | China, Yunnan | N/A | N/A | Ding,Y.-R., et al. |
| CN-YN3 | MG816566.1 | China, Yunnan | N/A | N/A | Ding,Y.-R., et al. |
| CN-YN4 | MG816565.1 | China, Yunnan | N/A | N/A | Ding,Y.-R., et al. |
| CN-YN5 | MG816564.1 | China, Yunnan | N/A | N/A | Ding,Y.-R., et al. |
| CN-YN6 | MG816563.1 | China, Yunnan | N/A | N/A | Ding,Y.-R., et al. |
| CN-YN7 | MG816562.1 | China, Yunnan | N/A | N/A | Ding,Y.-R., et al. |
| CN-YN8 | MG816561.1 | China, Yunnan | N/A | N/A | Ding,Y.-R., et al. |
| CN-YN9 | MG816560.1 | China, Yunnan | N/A | N/A | Ding,Y.-R., et al. |
| CN-YN10 | MG816559.1 | China, Yunnan | N/A | N/A | Ding,Y.-R., et al. |
| CN-YN11 | MG816558.1 | China, Yunnan | N/A | N/A | Ding,Y.-R., et al. |
| CN-YN12 | MG816557.1 | China, Yunnan | N/A | N/A | Ding,Y.-R., et al. |
| CN-CQ1 | MG816556.1 | China, Chongqing | N/A | N/A | Ding, Y.-R., et al. |
| CN-CQ2 | MG816555.1 | China, Chongqing | N/A | N/A | Ding, Y.-R., et al. |
| CN-CQ3 | MG816554.1 | China, Chongqing | N/A | N/A | Ding, Y.-R., et al. |
| CN-CQ4 | MG816553.1 | China, Chongqing | N/A | N/A | Ding, Y.-R., et al. |
| CN-CQ5 | MG816552.1 | China, Chongqing | N/A | N/A | Ding, Y.-R., et al. |
| CN-CQ6 | MG816551.1 | China, Chongqing | N/A | N/A | Ding, Y.-R., et al. |
| CN-CQ7 | MG816550.1 | China, Chongqing | N/A | N/A | Ding, Y.-R., et al. |
| CN-CQ8 | MG816549.1 | China, Chongqing | N/A | N/A | Ding, Y.-R., et al. |
| CN-CQ9 | MG816548.1 | China, Chongqing | N/A | N/A | Ding, Y.-R., et al. |
| CN-CQ10 | MG816547.1 | China, Chongqing | N/A | N/A | Ding, Y.-R., et al. |
| CN-CQ11 | MG816546.1 | China, Chongqing | N/A | N/A | Ding, Y.-R., et al. |
| CN-AH1 | MG816544.1 | China, Anhui | N/A | N/A | Ding,Y.-R., et al. |
| CN-AH2 | MG816543.1 | China, Anhui | N/A | N/A | Ding,Y.-R., et al. |
| CN-AH3 | MG816542.1 | China, Anhui | N/A | N/A | Ding,Y.-R., et al. |
| CN-AH4 | MG816541.1 | China, Anhui | N/A | N/A | Ding,Y.-R., et al. |
| CN-AH5 | MG816540.1 | China, Anhui | N/A | N/A | Ding,Y.-R., et al. |
| CN-AH6 | MG816539.1 | China, Anhui | N/A | N/A | Ding,Y.-R., et al. |
| CN-AH7 | MG816538.1 | China, Anhui | N/A | N/A | Ding,Y.-R., et al. |
| CN-AH8 | MG816537.1 | China, Anhui | N/A | N/A | Ding,Y.-R., et al. |
| CN-AH9 | MG816536.1 | China, Anhui | N/A | N/A | Ding,Y.-R., et al. |
| CN-AH10 | MG816535.1 | China, Anhui | N/A | N/A | Ding,Y.-R., et al. |
| CN-AH11 | MG816534.1 | China, Anhui | N/A | N/A | Ding,Y.-R., et al. |
| CN-AH12 | MG816533.1 | China, Anhui | N/A | N/A | Ding,Y.-R., et al. |

KR-YC, Yeoncheon (South Korea); KR-IC, Incheon (South Korea); KR-GR, Guryongpo (South Korea); CN-YN, Yunnan (China); Yunnan; CN-CQ, Chongqing (China); CN-AH, Anhui (China); TH-CM, Chiang Mai (Thailand); JP-NS, Nagasaki (Japan). N/A, no data.
