# Supplementary material for: Population genetic structure and evolutionary genetics of Anopheles sinensis based on knockdown resistance (kdr) mutations and mtDNA-COII gene in China–Laos, Thailand–Laos, and Cambodia–Laos borders
Source: Parasit Vectors. 2022 Jun 26;15:229. doi: 10.1186/s13071-022-05366-9 (PMC9233850; doi:10.1186/s13071-022-05366-9)
Supplement: Supplementary file 3 — Additional file 3: Table S3. a Genetic diversity indices and neutrality tests (Fu’s Fs and Tajima’s D) based on the kdr intron of An. sinensis. b Genetic diversity indices and neutrality tests (Fu’s Fs and Tajima’s D) based on the COII gene of An. sinensis. nd not determined; #P < 0.10; *P < 0.05; **P < 0.02; ***P < 0.001. n number of sequences; s number of polymorphic sites; pi nucleotide diversity; h number of haplotypes; Hd haplotype diversity; LPY Yot Ou County (Phongsaly Province); LXP Pak lay County (Xayabuli Province); LCP Pathoomphone County (Champasak Province); CN-AH Anhui Province (China); CN-GX Guangxi Province (China); CN-YN Yunnan Province (China); CN-HaN Hainan Province (China); CN-HeN Henan Province (China); CN-ZJ Zhejiang Province (China). [file 13071_2022_5366_MOESM3_ESM.docx]

**Table S3a. Genetic diversity indices and neutrality tests (Fu’s *Fs* and Tajima’s *D*) based on *kdr* intron of *An. sinensis***

| **Species** | **n** | **Haplotype Code** | **S** | **Pi** | **h** | **Hd** | k | Fu's Fs | Tajima's D |
| --- | --- | --- | --- | --- | --- | --- | --- | --- | --- |
| Total | 154 | H1(30), H2(20), H3(58), H4(16), H5(4), H6(13), H7(1), H8(1), H9(1), H10(5), H11(2), H12(1), H13(1), H14(1) | 10 | 0.02190 | 14 | 0.788 | 1.40183 | -5.17700^*^ | -0.51441 |
| LPY | 83 | H1(26), H2(14), H3(40), H4(2), H5(1) | 3 | 0.01333 | 5 | 0.648 | 0.85336 | -0.12200 | 0.77921 |
| LXP | 2 | H2(1), H6(1) | 1 | 0.01563 | 2 | 1.000 | 1.00000 | 0.00000 | n.d. |
| LCP | 4 | H96(4) | 0 | 0.00000 | 1 | 0.000 | n.d. | n.d. | n.d. |
| CN-AH | 10 | H3(4), H4(1), H5(3), H6(1), H13(1) | 3 | 0.02118 | 5 | 0.800 | 1.35556 | -1.50700^#^ | 1.00120 |
| CN-GX | 22 | H1(2), H2(3), H3(4), H4(4), H6(3), H7(1), H8(1), H9(1), H10(2), H11(1) | 8 | 0.02936 | 10 | 0.913 | 1.87879 | -4.51700^**^ | -0.47237 |
| CN-YN | 5 | H3(1), H4(1), H6(1), H10(2) | 4 | 0.02813 | 4 | 0.900 | 1.80000 | -1.19500^#^ | -0.41017 |
| CN-HaN | 10 | H2(1), H3(1), H4(4), H6(1), H10(1), H11(1), H14(1) | 6 | 0.02778 | 7 | 0.867 | 1.77778 | -3.66700^**^ | -0.66931 |
| CN-HeN | 8 | H1(2), H3(4), H64(1), H6(1) | 3 | 0.01953 | 4 | 0.750 | 1.25000 | -0.78500 | 0.33065 |
| CN-ZJ | 10 | H2(1), H3(4), H4(3), H6(1), H12(1) | 4 | 0.02569 | 5 | 0.800 | 1.64444 | -1.05200 | 0.62589 |

n.d., not determined; #, *P* < 0.10; *, *P* < 0.05; **, *P* < 0.02; ***, *P* < 0.001. Abbreviations: n, number of sequences; S, number of polymorphic sites; pi, nucleotide diversity; h, number of haplotypes; Hd, haplotype diversity; LPY, Yot Ou County (Phongsaly Province); LXP, Pak lay County (Xayabuli Province); LCP, Pathoomphone County (Champasak Province); CN-AH, Anhui Province (China); CN-GX, Guangxi Province (China); CN-YN, Yunnan Province (China); CN-HaN, Hainan Province (China); CN-HeN, Henan Province (China); CN-ZJ, Zhejiang Province (China).

**Table S3b. Genetic diversity indices and neutrality tests (Fu’s *Fs* and Tajima’s *D*) based on COII gene of *An. sinensis***

| **Species** | **n** | **Haplotype Code** | **S** | **Pi** | **h** | **Hd** | **k** | **Fu's *Fs*** | **Tajima's *D*** |
| --- | --- | --- | --- | --- | --- | --- | --- | --- | --- |
| Total | 89 | H1(13), H2(1), H3(2), H4(37), H5(1), H6(7), H7(2), H8(1), H9(1), H10(4), H11(1), H12(5), H13(1), H14(2), H15(1), H16(1), H17(1), H18(1), H19(1), H20(1), H21(3), H22(1) | 26 | 0.00351 | 22 | 0.799 | 2.38381 | -11.48900^***^ | -1.79501^**^ |
| LPY | 83 | H1(12), H3(2), H4(37), H5(1), H6(7), H7(2), H8(1), H9(1), H10(4), H11(1), H12(5), H13(1), H14(2), H15(1), H16(1), H17(1), H18(1), H19(1), H20(1) | 19 | 0.00282 | 20 | 0.783 | 1.92536 | -11.80300^***^ | -1.61923^*^ |
| LXP | 2 | H1(1), H2(1) | 5 | 0.00731 | 2 | 1.000 | 5.00000 | 1.60900 | n.d. |
| LCP | 4 | H21(3), H22(1) | 16 | 0.01175 | 2 | 0.500 | 8.00000 | 5.10000 | -0.84903^#^ |

n.d., not determined; #, *P* < 0.10; *, *P* < 0.05; **, *P* < 0.02; ***, *P* < 0.001. Abbreviations: n, number of sequences; S, number of polymorphic sites; pi, nucleotide diversity; h, number of haplotypes; Hd, haplotype diversity; LPY, Yot Ou County (Phongsaly Province); LXP, Pak lay County (Xayabuli Province); LCP, Pathoomphone County (Champasak Province).
