# Supplementary figures and images for: Population genetic structure and evolutionary genetics of Anopheles sinensis based on knockdown resistance (kdr) mutations and mtDNA-COII gene in China–Laos, Thailand–Laos, and Cambodia–Laos borders
Source: Parasit Vectors. 2022 Jun 26;15:229. doi: 10.1186/s13071-022-05366-9 (PMC9233850; doi:10.1186/s13071-022-05366-9)

a

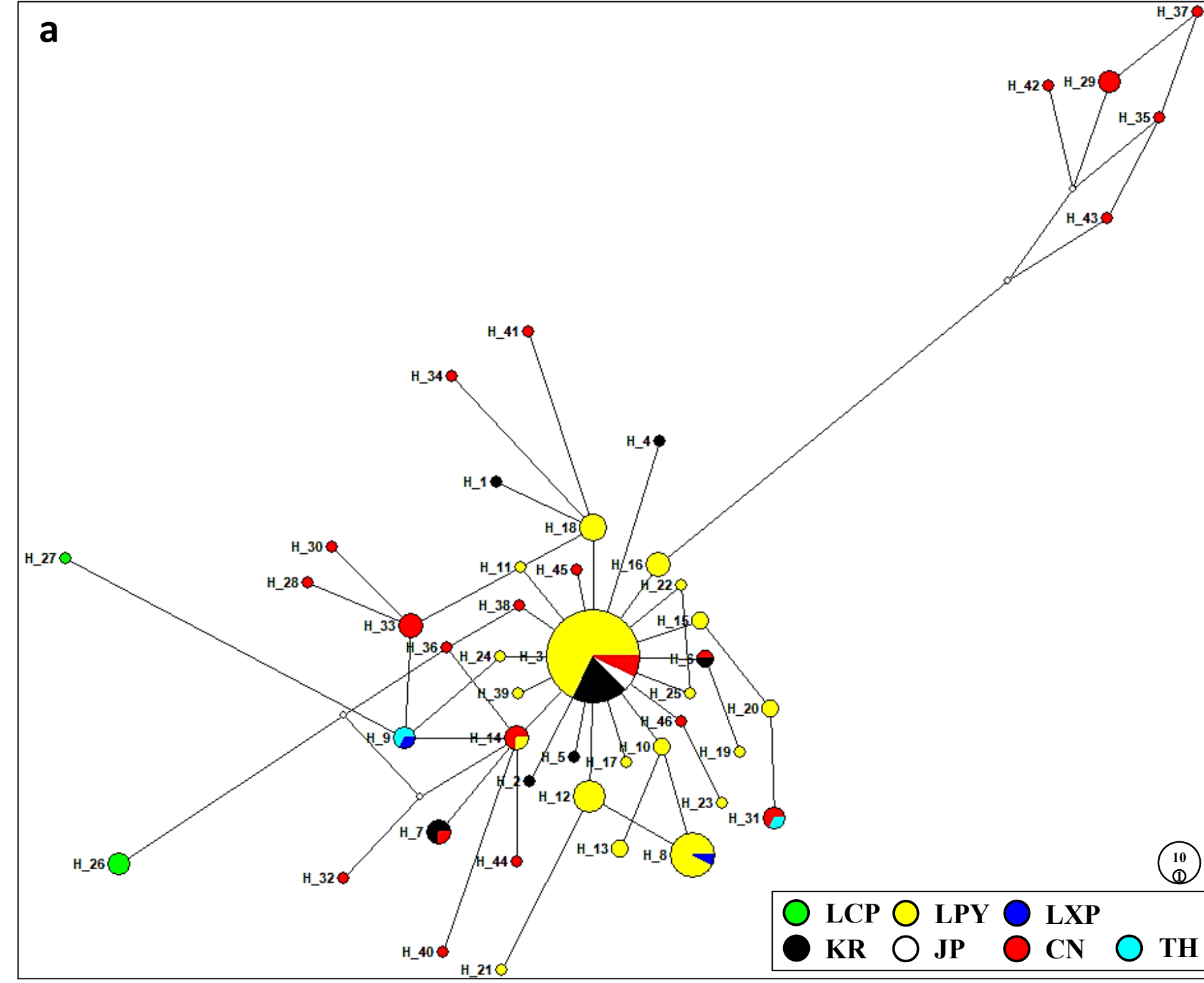

b

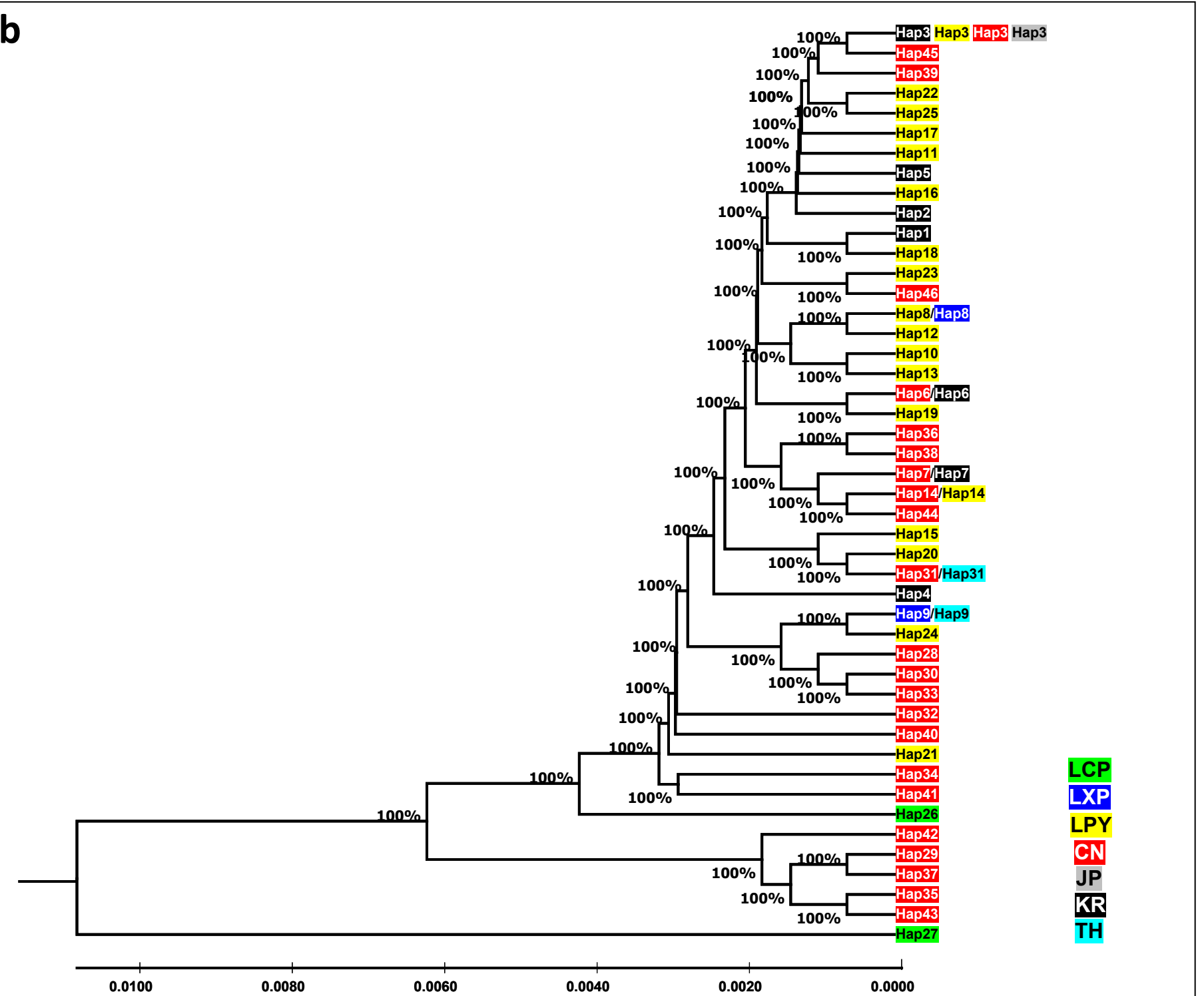

Supplement: Supplementary file 4 — Additional file 4: Figure S1. Phylogenetic analysis based on the COII sequences in An. sinensis populations from Laos and other countries. a Phylogenetic network of 46 mitochondrial haplotypes of the COII gene in An. sinensis. Localities are indicated by different colors (bottom right). The size of each circle is proportional to its corresponding frequencies. b UPGMA dendrogram based on Nei’s unbiased genetic distance between the 46 haplotypes of An. sinensis. Yellow, green, and blue circles/rectangles represent haplotypes found in this study from LPY (Phongsaly Province: Yot Ou County), LCP (Champasak Province: Pathoomphone County), and LXP (Xayabuli Province: Pak lay County) populations, respectively. Black, white, red, and sky blue circles/rectangles represent haplotypes found in NCBI data from Korea, Japan, China, and Thailand (Tables S2), respectively. [file 13071_2022_5366_MOESM4_ESM.pdf]

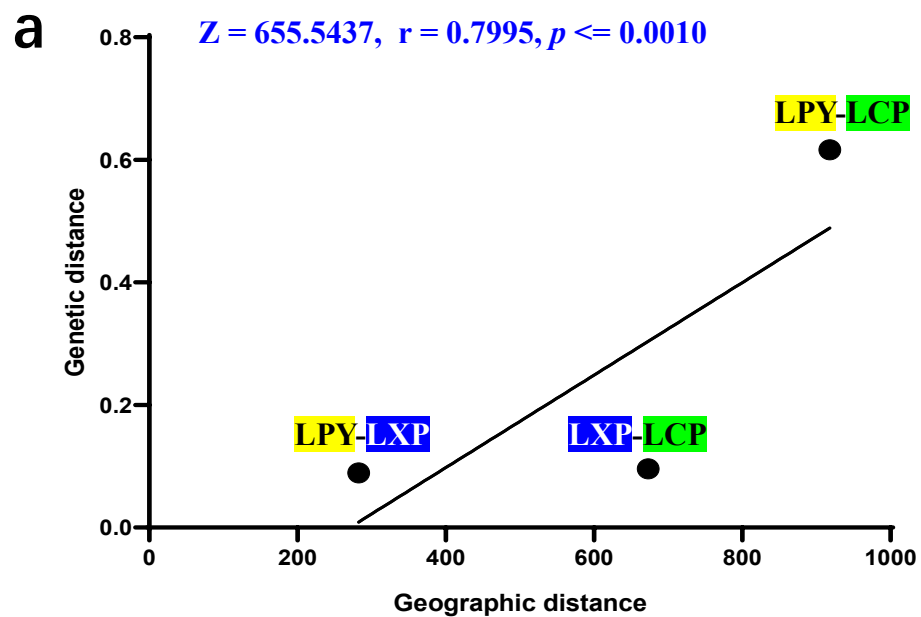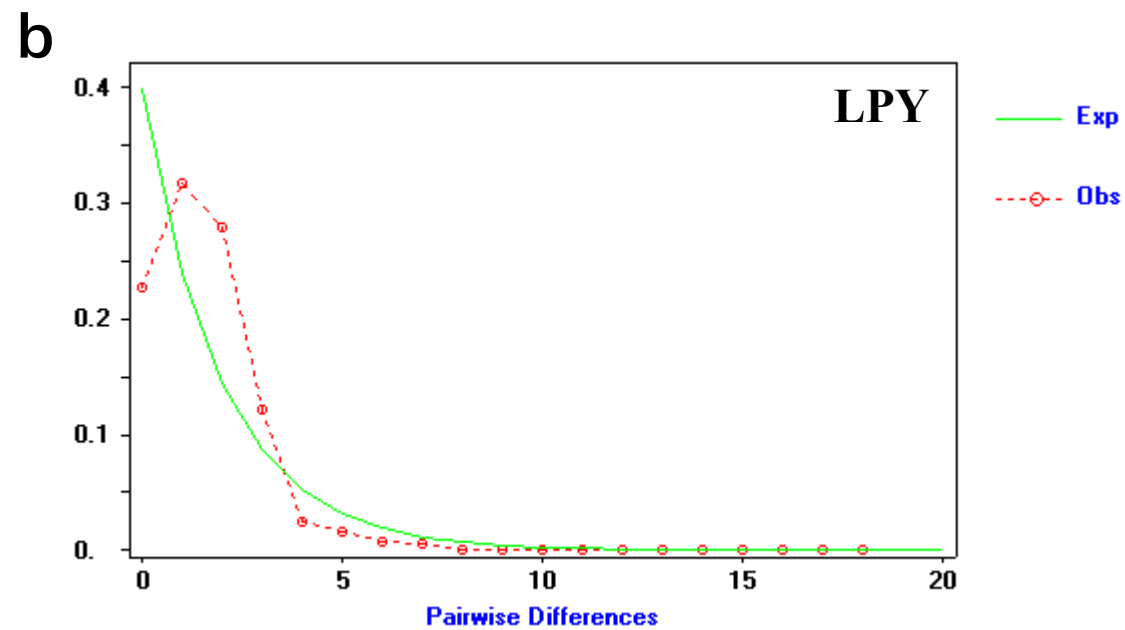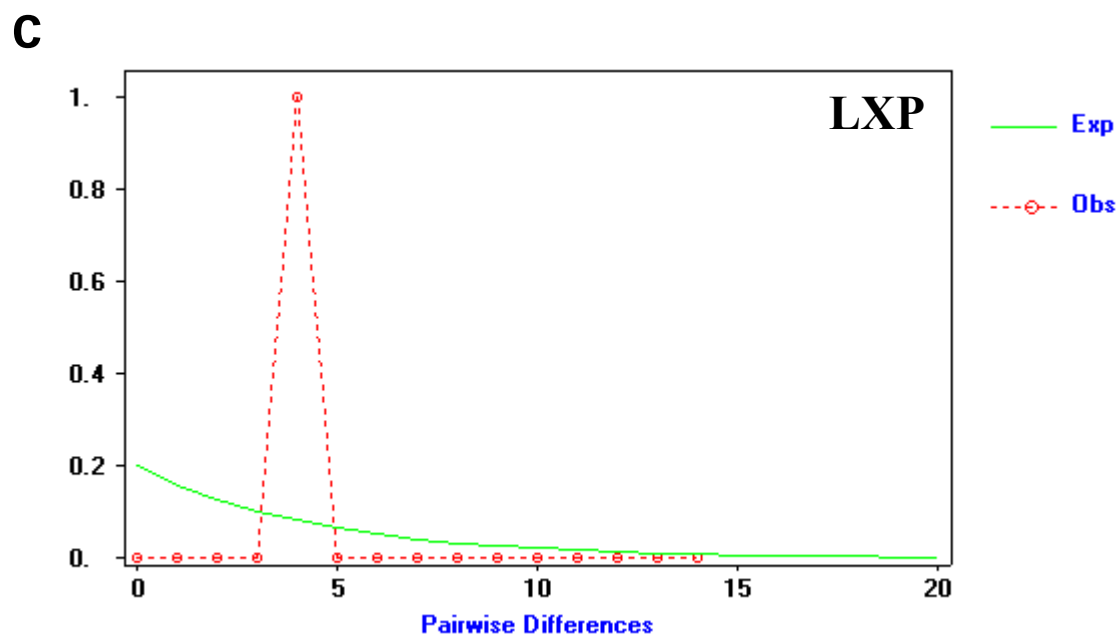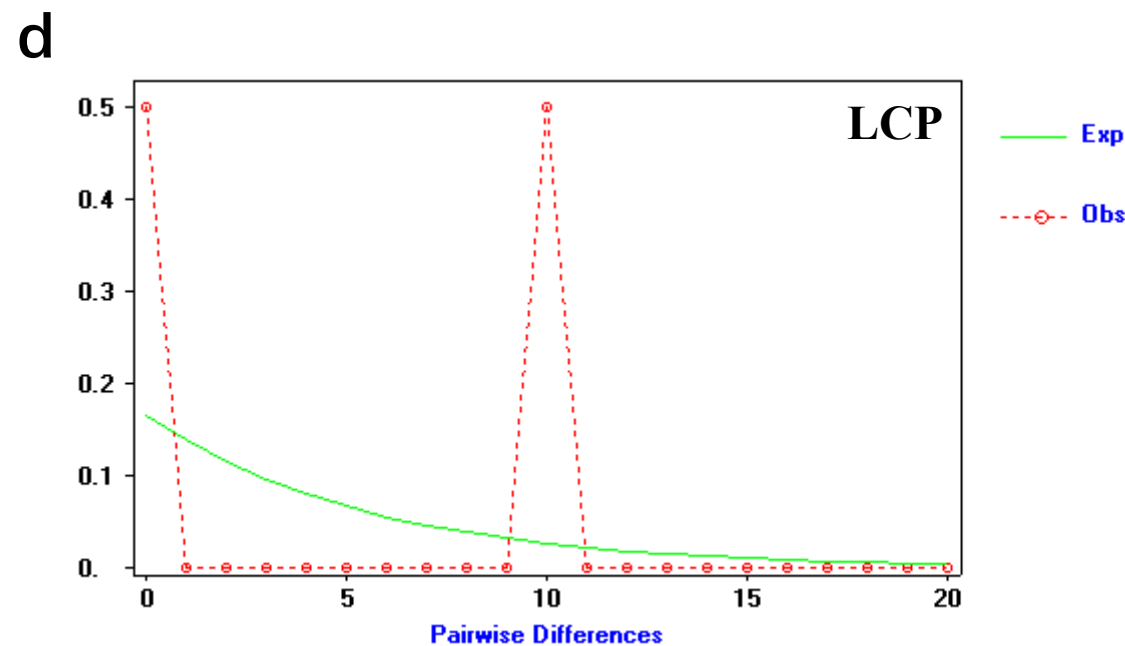

Supplement: Supplementary file 7 — Additional file 7: Figure S2. a Isolation by distance; the relationship between geographical and genetic distances based on COII sequences in An. sinensis populations. Isolation by distance (IBD) was examined using a nonparametric Mantel test with the web-based computer program IBDWS v.3.16. b–d Mismatch distribution graphs for An. sinensis population based on COII sequences. The x- and y-axis show the number of pairwise differences and the frequency of the pairwise comparisons, respectively. The observed frequencies are represented by a dotted line. The frequency expected under the hypothesis of the constant population model is depicted by a solid line. LPY, Yot Ou County, Phongsaly Province; LXP, Pak lay County, Xayabuli Province; LCP, Pathoomphone County, Champasak Province. [file 13071_2022_5366_MOESM7_ESM.pdf]
