# Supplementary material for: Population genetic structure and evolutionary genetics of Anopheles sinensis based on knockdown resistance (kdr) mutations and mtDNA-COII gene in China–Laos, Thailand–Laos, and Cambodia–Laos borders
Source: Parasit Vectors. 2022 Jun 26;15:229. doi: 10.1186/s13071-022-05366-9 (PMC9233850; doi:10.1186/s13071-022-05366-9)
Supplement: Supplementary file 5 — Additional file 5: Table S4. Analysis of molecular variance (AMOVA) of 10 An. sinensis populations based on COII. FCT, fixation index among groups; FSC, among populations within groups; FST, within populations. [file 13071_2022_5366_MOESM5_ESM.docx]

**Table S4. Analysis of molecular variance (AMOVA) of ten *An. sinensis* populations based on COII**

| **Source of variation** | **d. f.** | **Sum of squares** | **Variance components** | **% of variation** | **Fixation index (*P*)** |
| --- | --- | --- | --- | --- | --- |
| Among groups | 1 | 13.576 | 1.57049 Va | 58.43 | *F*_CT_: 0.06357 (*P*>0.05) |
| Among populations within groups | 1 | 1.324 | 0.07103 Vb | 2.64 | *F*_SC_: 0.61071 (*P>*0.05) |
| Within populations | 86 | 89.988 | 1.04637 Vc | 38.93 | *F*_ST_: 0.58428 (*P*<0.05) |
| Total | 88 | 104.888 | 2.68789 |  |  |

*F*_CT_, Fixation index among groups; *F*_SC_, among populations within groups; *F*_ST_, within populations.
