# Supplementary material for: Population genetic structure and evolutionary genetics of Anopheles sinensis based on knockdown resistance (kdr) mutations and mtDNA-COII gene in China–Laos, Thailand–Laos, and Cambodia–Laos borders
Source: Parasit Vectors. 2022 Jun 26;15:229. doi: 10.1186/s13071-022-05366-9 (PMC9233850; doi:10.1186/s13071-022-05366-9)
Supplement: Supplementary file 6 — Additional file 6: Table S5. Genetic differentiation and gene flow among the geographical groups of An. sinensis based on COII. The pairwise FST values and Nm values based on the COII are shown below and above the diagonal, respectively. Characters in bold indicated the significance (P < 0.05). Inf, infinite. LPY, Yot Ou County, Phongsaly Province; LXP, Pak lay County, Xayabuli Province; LCP, Pathoomphone County, Champasak Province. [file 13071_2022_5366_MOESM6_ESM.docx]

**Table S5. Genetic differentiation and gene flow among the geographic groups of *An. sinensis* based on COII**

|  | LPY | LXP | LCP |
| --- | --- | --- | --- |
| LPY |  | 5.10835 | 0.31093 |
| LXP | 0.08915 |  | 4.72727 |
| LCP | **0.61657** | 0.09565 |  |

The pairwise *F*_ST_ values and Nm values based on the COII are shown below and above the diagonal, respectively. **Characters** in bold indicated the significance (*P*<0.05). inf, infinite. LPY, Yot Ou County, Phongsaly Province; LXP, Pak lay County, Xayabuli Province; LCP, Pathoomphone County, Champasak Province.
